# Unveiling potent xanthine oxidase inhibitors in two *Balanophora* spp. using machine learning-based virtual screening and molecular docking approach

**DOI:** 10.1038/s41598-025-32282-6

**Published:** 2025-12-16

**Authors:** Nguyen Ngoc An, Dao Quang Tung, Le Van Tue, Nguyen Thanh Son, Nguyen Thanh Tung, Huong-Giang Le, Thai Chinh Tam, Nguyen Thị Thuan, Daniel Baecker, Do Thi Mai Dung

**Affiliations:** 1 VNU University of Engineering and Technology, 144 Xuan Thuy, Cau Giay, Hanoi, 100000 Vietnam; 2 Department of Computer and Systems Sciences, Stockholm University, 106 91 Stockholm, Sweden; 3 Hanoi University of Pharmacy, 13 - 15 Le Thanh Tong, Cua Nam, Hanoi, 100000 Vietnam; 4 Department of Pharmacognosy and Traditional Pharmacy, School of Pharmacy, University of Medicine and Pharmacy at Ho Chi Minh City, Ho Chi Minh City, 700000 Vietnam; 5 Department of Pharmaceutical and Medicinal Chemistry, Institute of Pharmacy, Freie Universität Berlin, Königin-Luise-Straße 2+4, 14195 Berlin, Germany; 6 Unit of Computation and AI Application Research, Faculty of Pharmaceutical Chemistry and Technology , Hanoi University of Pharmacy, 13-15 Le Thanh Tong, Cua Nam, Hanoi, 100000, Vietnam

**Keywords:** *Balanophora* species, Xanthine oxidase inhibitors, Machine learning, Docking, XGBoost, Computational biology and bioinformatics, Drug discovery

## Abstract

Pharmacological studies revealed that the *Balanophora* species contains diverse phytochemicals which enable interesting biological activities and emphasize their pharmaceutical relevance. Previously, we identified significant xanthine oxidase (XO) inhibitory activity from extracts of the two *Balanophora* spp. (*Balanophora subcupularis* P.C. Tam and *Balanophora tobiracola* Makino). However, the specific compounds responsible for this activity remain unidentified so far. Thus, in the present study, we focused on elucidating the compounds inducing the XO inhibitory effect of extracts from *Balanophora* species. Therefore, a combination of advanced liquid chromatography and mass spectrometry (LC-QToF-HRMS), virtual screening using machine learning (ML) models, and molecular docking simulation was applied. Using LC-QToF-HRMS, 23 and 21 compounds were identified in the ethyl acetate fractions of *B. subcupularis* and *B. tobiracola*, respectively. Next, a curated dataset of natural and synthetic compounds with known XO inhibitory activity was employed to train several ML models. Adducing five selected ML models, the virtual screening process identified the potentially active compounds 1-(3,4-dihydroxyphenyl)-6,7-dihydroxy-1,2-dihydro-2,3-naphthalenedicarboxylic acid, taxifolin, and 1-*O*-caffeoyl-6-*O*-(S)-brevifolincarboxyl-*β*-D-glucopyranose. All the compounds found in the two *Balanophora* spp. underwent docking simulations, in which MTE, FES, and AFH were retained in the active site of XO, ensuring reliable re-docking results. Finally, taxifolin emerged as the most promising novel XO inhibitor, demonstrating greater potential than the established drug allopurinol, as supported by both the virtual screening nomination and docking simuation. These findings contribute to the development of natural XO inhibitors and may open new opportunities for gout treatment and uric acid level control.

**Supplementary Information:**

The online version contains supplementary material available at 10.1038/s41598-025-32282-6.

## Introduction

The genus *Balanophora* J. R. Forst. & G. Forst., belonging to the family *Balanophoraceae*, comprises approximately 23 species of parasitic plants predominantly distributed in Asia, Africa, and Australia. These plants are holoparasites that rely entirely on host plants for nutrients and are characterized by their highly reduced morphologies and unique reproductive structures^1^. Traditionally, *Balanophora* species have been widely utilized in various Asian medicinal practices for treating ailments such as stomach pain, uterine prolapse, wounds, hemorrhoids, and inflammation. They have also been employed due to hemostatic, antipyretic, and analgesic properties^2,3^. For instance, in Chinese medicine and Vietnamese folk medicine, *Balanophora fungosa* is particularly valued for its ability to improve blood circulation, reduce swelling, and promote wound healing^4^. In Taiwan, *Balanophora laxiflora* Hemsl. has been used as a medicinal plant to treat cough, metrorrhagia, and hemorrhoids^5^.

Pharmacological studies have revealed that these plants contain diverse phytochemicals, including tannins, flavonoids, lignans, terpenes, and phenylpropanoids, which contribute to their biological activities^6^. Notably, *Balanophora* extracts demonstrated potent antioxidant and anti-inflammatory effects, making them promising candidates for combating oxidative stress-related diseases^7,8^. They also exhibited antimicrobial activity against various bacterial strains, indicating their potential as natural antibiotics^9^. Some species, such as *Balanophora polyandra* and *Balanophora japonica*, showed cytotoxicity against cancer cell lines, suggesting anticancer potential through induction of apoptosis and cell cycle arrest^10,11^. Furthermore, *Balanophora* species displayed hypouricemic effects by inhibiting xanthine oxidase (XO), a key enzyme involved in uric acid production, offering a natural alternative for managing gout and hyperuricemia. Compounds such as hydrolysable tannins were identified as effective XO inhibitors, rivaling the potency of synthetic drugs like allopurinol^5^. Other pharmacological properties of *Balanophora* extracts include hepatoprotective effects, neuroprotection, gastroprotection, and enhanced inhibition of melanin synthesis, making these plants valuable for cosmetic and dermatological applications^6^. This growing body of evidence highlights *Balanophora* as a promising genus for drug discovery and supports its integration into modern medicine while preserving its traditional use​. Despite their diverse bioactivity potentials, further research is required to explore their mechanisms of action, toxicological profiles, and clinical applications.

Our research focuses on two relatively understudied species within this genus, i.e., *Balanophora subcupularis* and *Balanophora tobiracola*. The existing literature on these species is sparse, with few studies detailing their phytochemical composition or pharmacological activities. Notably, *B. tobiracola* was found to contain hydrolysable tannins, particularly ellagitannins, which exhibited radical-scavenging and potential HIV-inhibiting activites^12^. Related studies on *B. japonica* reported on the presence of bioactive caffeoyl and galloyl derivatives with antioxidant and enzyme-inhibitory activities on α-glucosidase^13^. Previous investigations by our group identified significant XO inhibitory activity in extracts from both species, with the ethyl acetate fraction demonstrating the most potent effect^14^. However, the specific compounds responsible for this activity remain unidentified thus far. In general, XO is an enzyme catalyzing the oxidation of xanthine and hypoxanthine to uric acid. In the active center, XO bears two flavin adenine dinucleotides, twice molybdenum and eight-times iron. The enzyme is involved in the production of reactive oxygen species (ROS) and thus contributes to oxidative stress^15^. Its increased activity causes enhanced levels of uric acid and thus contributes to the development of gout and other hyperuricemia-related disorders^16^. Hence, inhibition of XO with small molecules (e.g., allopurinol) is a main strategy to treat such diseases^17^.

To address this, we employed advanced liquid chromatography coupled with quadrupole time-of-flight high-resolution mass spectrometry (LC-QToF-HRMS) to comprehensively profile the chemical constituents present in the ethyl acetate extracts. LC-QToF-HRMS represents a state-of-the-art analytical platform for comprehensive phytochemical profiling. Its exceptional sensitivity and mass accuracy facilitate precise molecular formula determination, enabling the identification of both known and novel compounds within complex plant extracts. Furthermore, its advanced fragmentation capabilities provide detailed structural insights, allowing differentiation of isomers and closely related compounds. The suitability of the technique for non-targeted metabolomics and broad-spectrum compound detection underscores its critical role in natural product research and drug discovery^18^.

Despite enormous advancement in characterization of phytochemicals and pharmacological screening across botanical sources, activity-oriented isolation methods still have a number of shortcomings. These methods are time-consuming, costly, and are mainly concerned with the isolation of separate components, but the discovered biological activity of natural constituents often corresponds to the resultant of synergetic interactions among various molecules. In addition, the elucidation of structure and verification of activity of low-abundance or unstable components are difficult and thus complicate the whole research process. In this context, both *B. tobiracola* and *B. subcupularis* are demonstrably rare in the wild and conservation-sensitive, with few documented populations and restricted distributions^19,20^. Because activity-guided isolation requires considerable biomass for repeated fractionation and assays, it may exert disproportionate pressure on these already limited populations. In contrast, machine learning (ML)-based screening provides a modern, data-driven alternative by allowing the extraction of patterns from existing chemical and biological data to predict the potential bioactivity of compounds. Instead of isolating and testing individual components, ML models can learn meaningful representations of molecular structures to predict inhibitory potential or other bioactivities. This approach shortens time and cost for testing and increases the screening range to difficult-to-isolate or first-test-before components. Recent works have shown that combining chemical and biological data sets and ML-based models has been able to evidently enhance predictive accuracy and generalizability and provide a more efficient route to natural product discovery^21^. Within efforts to discover XO inhibitors, Wu et al. developed a ML-assisted quantitative structure–activity relationship (QSAR) model that effectively predicted the inhibitory potency from molecular fingerprints^22^. Similarly, Zhou et al. combined ML approaches with molecular simulations to screen natural compounds for XO inhibitory activity, successfully identifying vanillic acid as a promising XO inhibitor candidate^23^. However, existing ML approaches often face challenges such as small training datasets^24,^ lack of applicability domain (AD) definitions^25^, and reduced prediction reliability when applied to novel chemical spaces. To address these limitations, we expanded our dataset by integrating diverse compound libraries and established robust applicability domains to enhance prediction confidence. This strategic improvement ensures that models can generalize effectively and provide accurate predictions for new compounds.

This study focuses on elucidating the compounds causing the XO inhibitory effect of two *Balanophora* spp. extracts through a systematic combination of advanced liquid chromatography, LC-QToF-HRMS, virtual screening using ML models, and molecular docking simulation. The results highlight the potential of *Balanophora* species as sources of natural XO inhibitors and is hoped to provide a framework for developing safer and more effective therapeutic options to manage hyperuricemia and gout.

## Methods

### Identification of chemical constituents

#### Plant material

Fresh plant materials of *Balanophora subcupularis* (BS, 2.5 kg) were collected in Muong Lay District, Dien Bien Province, Vietnam (22°03′56″ N; 103°06′13″ E) in November 2017.

Samples of *Balanophora tobiracola* (BT, 3.0 kg) were collected in Bac Son District, Lang Son Province, Vietnam (21°53′33″ N; 106°22′57″ E) in January 2018. Voucher specimens were deposited at the Department of Botany, Hanoi University of Pharmacy (*B. subcupularis*: HNIP/18,638/21*; B. tobiracola*: HNIP/18,640/21), the Department of Plant Resources, Institute of Ecology and Biological Resources (IEBR/TNTV-03 and IEBR/TNTV-07), and the Faculty of Biology, VNU University of Science, Vietnam National University, Hanoi (HNU 024,068 and HNU 024,056). The botanical identification of both species was performed by Dr. Nguyen Quang Hung (Department of Plant Resources, Institute of Biology, Vietnam Academy of Science and Technology, Hanoi, Vietnam).

Fieldwork and collection of wild plant materials was carried out in accordance with the Vietnamese legislation on biodiversity conservation and the management of endangered forest plants, including the Law on Biodiversity (Law No. 20/2008/QH12 of the National Assembly of Vietnam) and its implementing regulations such as Governments Decree No. 06/2019/NĐ-CP and Decree No. 84/2021/NĐ-CP on the management of endangered, precious, and rare forest plants and animals as well as the implementation of the Convention on International Trade in Endangered Species of Wild Fauna and Flora (CITES). On the other hand, *B. subcupularis* and *B. tobiracola* are not listed as engangered species according to the current IUCN Red List and the CITES Appendices and are not included in the CITES Appendices. Therefore, this study complies with the IUCN Policy Statement on Research Involving Species at Risk of Extinction and with CITES.

#### Chemical components by LC-QToF-HRMS

For chromatographic analysis, 200 g of air-dried and powdered herbs of each species were extracted with 80% aqueous methanol (3 times) in a sonic bath. After filtration, the filtrate was evaporated *in vacuo*. The extract was then suspended in water and successfully partitioned with *n*-hexane (3 times) and ethyl acetate (3 times). The ethyl acetate fractions were evaporated *in vacuo* to obtain ethyl acetate extracts of *B. subcupularis* (8 g) and *B. tobiracola* (10 g)*.* The ethyl acetate extracts (10 mg) were then dissolved in methanol and transferred to a 5.0 mL volumetric flask, which was filled up with methanol. The mixtures were then filtered through a 0.45 µm syringe filter membrane and the filtrate were transferred into vials prior to analysis with LC-QToF-HRMS.

The liquid chromatographic analysis of the solutions was carried out using an Exion LC™ coupled to a X500R Q-TOF mass spectrometer (Sciex, USA). Separation of the compounds was performed with a Hypersil GOLD Dim. column (150 mm × 2.1 mm, 3 µm) (Thermo Scientific, USA). The flow rate from the delivery system was set at 0.400 mL/min, the sample injection volume was 2.0 µL. The mobile phase consisted of (A) 0.1% formic acid in water and (B) 0.1% formic acid in acetonitrile (Merck, Darmstadt, Germany). A linear gradient elution program was applied as follows: 0–1.0 min (0% B), 1.0–20.0 min (2% B), 20.0–25.0 min (98% B). MS/MS detection was performed in negative ion mode in the m/z interval of 50–2000 amu. Phenolic compounds were identified by mass to charge ratio (m/z), retention time, and MS fragmentation patterns. The identification was confirmed with commercial standards of gallic acid, *p*-coumaric acid, *trans*-caffeic acid, cinnamic acid, and kaempferol. Mass errors (Δppm) were computed from calibrated measurements versus theoretical [M ± adduct] masses and are reported to two decimal places. Values shown as 0.00 ppm reflect deviations < 0.005 ppm due to rounding, not absolute zero.

#### Chromatographic and spectral data processing and annotation

Raw LC–MS/MS data were processed in MZmine 2.33, with mass detection thresholds set at 200 (MS) and 20 (MS/MS). Chromatograms were generated using ions with a 0.02-min time span, ≥ 5000 peak height, and an m/z tolerance of 0.002 (5 ppm). Missing data were filled via the peak extender module, and chromatograms were deconvoluted employing a baseline cutoff algorithm. Aligned peak tables excluded peaks lacking MS/MS scans, filtered by the Global Natural Product Social Molecular Networking (GNPS) module, and gap-filled using the peak finder.

#### Molecular networking and annotation

Global Natural Product Social Molecular Networking (GNPS)^26^ generated molecular networks with edges retained for cosine similarity > 0.70 and ≥ 4 matched peaks (job ID: 23dfe918a19b41ed87b62b9786f68a38 (*B. subcupularis*); 0c3770afb4424203b66b44ecdb1f4c68 (*B. tobiracola*), obtained on May 20th of 2024 on https://gnps.ucsd.edu/). The spectra were queried against the GNPS spectral library and visualized with the software Cytoscape (version 3.10.2).

#### In silico annotation and integration

Network Annotation Propagation (NAP) annotated networks with top 10 candidate structures using a 5-ppm tolerance and the SuperNatural database. MS2LDA extracted Mass2Motifs using 5-ppm m/z and 10-s retention time tolerances. MolNetEnhancer integrated NAP and MS2LDA data, providing chemical class annotations and visualizing motif distributions.

### Machine learning model training

#### Data collection and molecular descriptor calculation

The dataset was compiled by collecting 625 XO inhibitory structures from research articles on CHEMBL33^27^. Molecular fingerprints, including MACCS-167 bits, ECFP4-1024 bits, ECFP4-2048 bits, ECFP6-1024 bits, and ECFP6-2048 bits, were generated for each compound using the RDKit toolkit^28^. During the preprocessing stage, compounds that could not be properly encoded were removed, resulting in a total of 483 compounds for further analysis.

To evaluate the structural diversity of the dataset, the Tanimoto coefficient (Tc)^29^ was used. The Tc is a widely recognized metric for assessing structural similarity between compound pairs and is calculated using formula (1):

1 $$Tc = \frac{{\left| {A \cap B} \right|}}{{\left| {A \cup B} \right|}}$$

where A and B represent the encoded molecular fingerprints of the compounds. In this study, Tc values were computed using ECFP4-1024 bit fingerprints. The value of the Tc can range from 0 to 1, where a value close to 1 indicates high structural similarity between two compounds, while a value closer to 0 indicates greater dissimilarity. By calculating the Tc for all compound pairs, this analysis provides insights into the structural variability within the dataset, ensuring a balanced representation of chemical space.

After preprocessed, the 483 compounds were divided randomly into three sets using the train_test_split function from sklearn library: training (70%), validation (15%), and test (15%). The splitting was done using a random shuffle to remove any possible bias from the original dataset order and provide an unbiased distribution. Stratified sampling was used to maintain the proportional representation of each class in all sets. This approach helped maintain dataset representativeness and prevent biases that could affect model training and evaluation. The validation set was used to select optimal hyperparameters, while the test set was employed to objectively evaluate the effectiveness of the model after hyperparameter optimization. The training set consisted of 150 active and 187 inactive compounds, the validation set contained 33 active and 40 inactive compounds, and the test set included 33 active and 40 inactive compounds. The total number of compounds in each dataset was 337 for training and 73 for validation and testing.

**Fig. 1:**
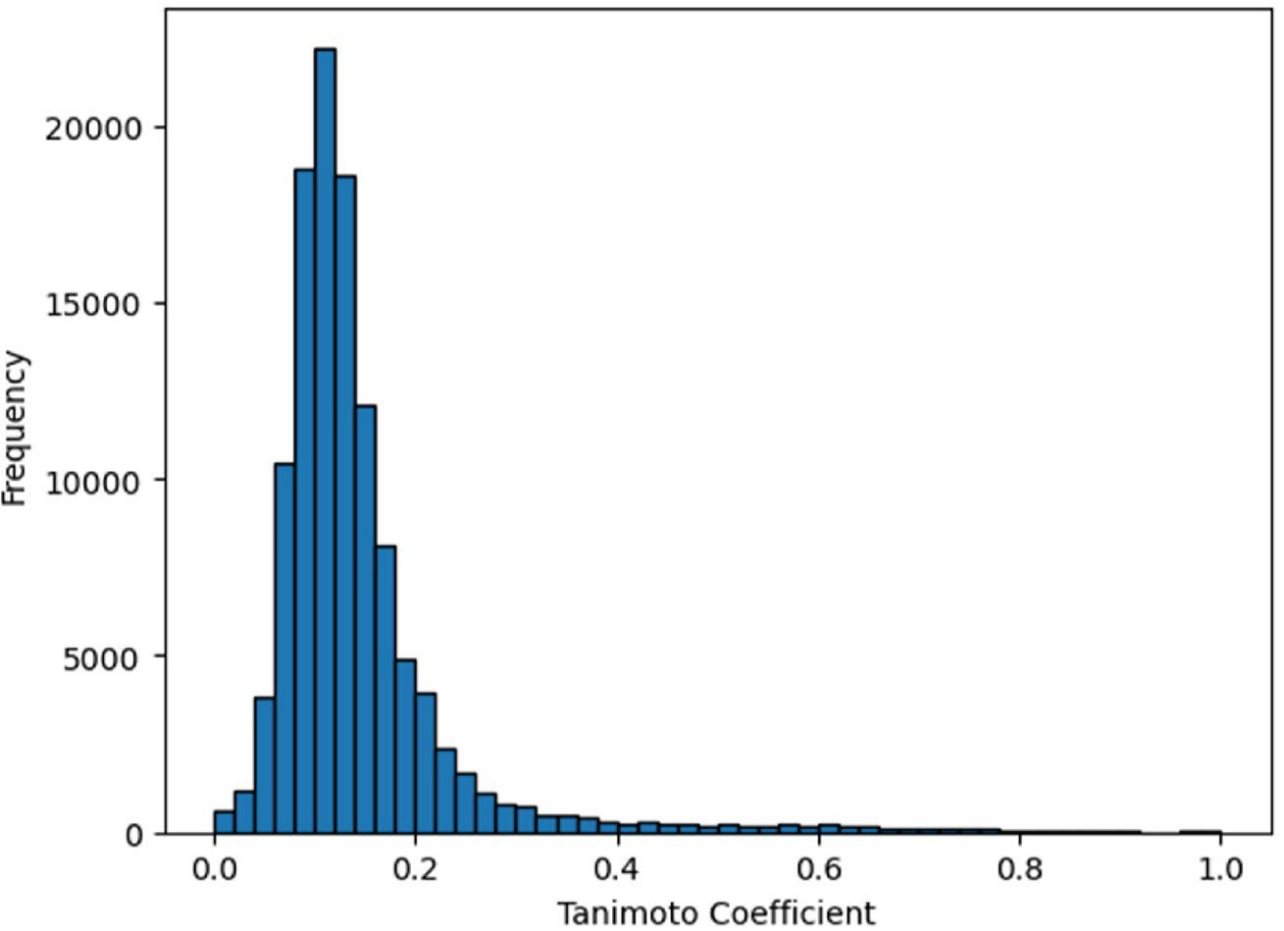
Pairwise values of the Tc distribution of the input dataset.

The structural diversity of the dataset was assessed by calculating the values of the Tc for all compound pairs. The results, presented in Fig. 1 and Table S1 (Supplementary Information), show that approximately 96.7% of the Tc for compound pairs encoded by the ECFP4-1024 bit algorithm is below 0.4, indicating that the dataset has relatively high structural diversity.

#### Performance assessment

All models were based on the Extreme Gradient Boosting (XGBoost) algorithm^30^, a decision trees algorithm using gradient boosting to improve performance. The XGBoost library with python programming language was used to implement and train models. The optimization of hyperparameters^31^ was processed to prevent overfitting and help the model achieve good prediction results. Specifically, the hyperparameters were optimized to maximize the predictive performance of the model while ensuring that the accuracy difference among the train, validation, and test sets did not exceed 5%, thereby maintaining generalization and preventing overfitting. The models were optimized for hyperparameters using the Grid Search method, with the search space consisting of the hyperparameters of the XGBoost algorithm as follows: the parameter “n_estimators” took values from 5 to 200; the parameter “max_depth” ranged from 2 to 10; the parameter “learning_rate” had one of the values 0.001, 0.01, 0.1; the parameter ‘colsample_bytree’ took values 0.5, 0.7, 0.9; the parameter ‘reg_lambda’ had one of the values 0, 0.001, 0.01, 0.1, 1; the parameter ‘min_child_weight’ was set to one of the values 7, 9, 11, 13.

By examining the label distribution, we observed a mild imbalance in the XO dataset (active ~ 45%, inactive ~ 55%), which limits potential training bias. To preserve the natural data distribution, we did not perform any explicit imbalance-handling techniques, such as oversampling or undersampling. Instead, rather than relying solely on accuracy (the proportion of correct predictions out of the total number of data), we reported multiple complementary metrics including precision, recall, F1-score, and area under the ROC curve (AUC) to provide a more comprehensive assessment. All metrics are computed under stratified tenfold cross-validation to preserve class proportions across folds^32^. The formulas for these metrics are shown in the following Eqs. (2) to (5)

2 $${\text{Accuracy }} = \frac{TP + TN}{{TP + TN + FP + FN}}$$

3 $${\text{F1}} - {\text{score }} = \frac{2TP}{{2TP + FP + FN}}$$

4 $${\text{Precision }} = \frac{TP}{{TP + FN}}$$

5 $${\text{Recall }} = \frac{TP}{{TP + FP}}$$

In the above formulas, TP, FP, TN, and FN represent the number of true positive, false positive, true negative, and false negative compounds, respectively.

### Virtual screening

Using LC-QToF-HRMS, 23 compounds were identified in the ethyl acetate fraction of *B. subcupularis* and 21 compounds in the ethyl acetate fraction of *B. tobiracola* (Tables 1 and 2). Prior to screening, all structures were rigorously analyzed to ascertain their compliance with the applicability domain of the predictive models. The dataset was preprocessed following a systematic workflow: (1) elimination of duplicate compounds; (2) calculation of molecular weight and extended-connectivity fingerprints (ECFP4) as 1024-bit vectors; and (3) dataset filtering based on two criteria: (3a) molecular weight within the range of 200–700 Da, and (3b) a mean Tc ≥ 0.4 for the compound compared to its five most similar compounds in the training set. Only compounds meeting these criteria were subsequently evaluated for potential XO inhibitory activity using all five models. From 33 compounds identified in the extracts of the two *Balanophora* spp., a total of 19 compounds met the criteria for the application domain, with 9 compounds from the ethyl acetate fraction of *B. subcupularis* and 10 compounds from that of *B. tobiracola*, collectively referred to as the screening set. Within this application domain, the trained models were expected to provide reliable predictions.

**Table 1:** The characterized metabolites originating from the ethyl acetate extract of *B. subcupularis.*

No	t_R_ (min)	Compound name	Ion adduct	Precursor/Product ion (m/z)	Molecular formula (error in ppm)	References
1	1.13	D-saccharose	[M−H]^−^	341.109 (59, 113, 164, 202, 244)	C_12_H_22_O_11_ (2.93)	^33^
2	1.17	D-trehalose	[M+HCOO]^−^	387.115 (59, 89, 119, 179, 341)	C_12_H_22_O_11_ (2.58)	^34^
3	1.35	malic acid	[2 M+Na−2H]^−^	289.018 (71, 115, 133)	C_4_H_6_O_5_ (3.46)	^35^
4	4.09	gallic acid	[M−H]^−^	169.014 (51, 79, 125)	C_7_H_6_O_5_ (0.00)	^36^
5	5.61	1,6-di-*O*-gallyol-β-D-glucose	[M−H]^−^	483.076 (169, 271, 331)	C_20_H_20_O_14_ (− 2.07)	^37^
6	6.25	strictinin	[M−H]^−^	633.073 (193, 300, 483)	C_27_H_22_O_18_ (0.00)	^38^
7	6.28	1-*O*-vanilloyl-β-D-glucose	[M−H]^−^	329.088 (59, 71, 89, 101, 151, 167, 209)	C_14_H_18_O_9_ (3.04)	^39^
8	7.57	1,3,6-tri-*O*-galloyl-β-D-glucose	[M−H]^−^	635.086 (169, 295, 483)	C_27_H_24_O_18_ (− 3.15)	^40^
9	8.21	1,2,4,6-tetra-*O*-galloyl-β-D-glucopyranoside	[M−H]^−^	787.099 (169, 295, 465, 635)	C_34_H_28_O_22_ (0.00)	^41^
10	8.34	6-*O*-[(2*E*)-3-(4-hydroxyphenyl)-2-propenoyl]-1-*O*-(3,4,5-trihydroxybenzoyl)hexopyranose	[M+Cl]^−^	477.103 (125, 169, 313)	C_22_H_22_O_12_ (0.00)	^42^
11	8.37	pyracanthoside	[M−H]^−^	449.109 (151, 287)	C_21_H_22_O_11_ (2.23)	^43^
12	8.47	lariciresinol-4-*O*-β-D-glucoside	[M−H]^−^	521.202 (175, 329)	C_26_H_34_O_11_ (0.00)	^44^
13	8.89	pentagalloyl glucose	[M−H]^−^	939.108 (769)	C_41_H_32_O_26_ (− 2.13)	^45^
14	9.19	1,6-di-*O*-galloyl-2-*O*-p-coumaroyl-β-D-glucose	[M−H]^−^	629.112 (169, 477)	C_29_H_26_O_16_ (− 3.18)	GNPS libraries
15	9.26	luteolin-7-*O*-glucoside	[M−H]^−^	447.092 (151, 285)	C_21_H_20_O_11_ (− 2.24)	^46^
16	9.41	(6*R*,7*R*,8*S*)-isolariciresinol	[M−H]^−^	359.15 (109, 159, 203, 241, 313, 344)	C_20_H_24_O_6_ (2.78)	^47^
17	9.56	ellagic acid	[M−H]^−^	300.998 (145, 185, 229)	C_14_H_6_O_8_ (0.00)	^48^
18	9.67	rosmarinic acid	[M−H]^−^	359.077 (72, 133, 161, 179)	C_18_H_16_O_8_ (0.00)	^47^
19	9.72	azelaic acid	[M−H]^−^	187.098 (57, 97, 125)	C_9_H_16_O_4_ (5.34)	^49^
20	9.98	phloretin	[M−H]^−^	273.076 (81, 167, 214)	C_15_H_14_O_5_ (0.00)	^50^
21	9.98	naringenin	[M−H]^−^	271.061 (65, 83, 119, 151, 177, 229)	C_15_H_12_O_5_ (0.00)	^51^
22	9.98	phloridizin	[M−H]^−^	435.128 (167, 273)	C_21_H_24_O_10_ (− 2.30)	^52^
23	10.10	1-*O*-(*E*)-cinnamoyl-4-galloyl-β-D-glucopyranose	[M−H]^−^	461.109 (125, 169, 211, 313, 401)	C_22_H_22_O_11_ (2.17)	GNPS libraries

**Table 2:** The characterized metabolites originated from the ethyl acetate extract of *B. tobiracola.*

No	t_R_ (min)	Compound name	Ion adduct	Precursor/Product ion (m/z)	Molecular formula (error in ppm)	References
1	4.49	strictinin	[M−H]^−^	633.073 (174, 300, 365, 404)	C_27_H_22_O_18_ (0.00)	^38^
2	5.96	1,6-di-*O*-gallyol-β-D-glucose	[M−H]^−^	483.077 (125, 169, 331)	C_20_H_20_O_14_ (0.00)	^37^
3	7.00	1-(3,4-dihydroxyphenyl)-6,7-dihydroxy-1,2-dihydro-2,3-naphthalenedicarboxylic acid	[M−H]^−^	357.062 (109, 159, 203, 269, 313)	C_18_H_14_O_8_ (2.80)	GNPS libraries
4	7.08	7-β-1-D-glucopyranosyl 11-methyl oleoside	[M−H]^−^	565.176 (59, 89, 223, 265)	C_23_H_34_O_16_ (− 1.77)	GNPS libraries
5	7.36	1,3,6-tri-*O*-galloyl-β-D-glucose	[M−H]^−^	635.088 (169, 295, 423, 483)	C_27_H_24_O_18_ (0.00)	^40^
6	7.49	secoxyloganin	[M−H]^−^	403.125 (59, 89)	C_17_H_24_O_11_ (2.48)	^53^
7	7.93	taxifolin	[M−H]^−^	303.051 (125, 175)	C_15_H_12_O_7_ (3.30)	^54^
8	8.33	lariciresinol-4-*O*-β-D-glucoside	[M−H]^−^	521.202 (89, 175, 329)	C_26_H_34_O_11_ (0.00)	^44^
9	8.35	1,2,4,6-tetra-*O*-galloyl-β-D-glucopyranoside	[M−H]^−^	787.098 (635)	C_34_H_28_O_22_ (− 1.27)	^41^
10	8.38	pyracanthoside	[M+Cl]^−^	485.085 (151, 287)	C_21_H_22_O_11_ (0.00)	^43^
11	8.56	luteolin-7-*O*-glucoside	[M−H]^−^	447.093 (151, 285)	C_21_H_20_O_11_ (0.00)	^46^
12	8.60	quercetin 7-*O*-β-D-glucopyranoside	[M−H]^−^	463.088 (301)	C_21_H_20_O_12_ (0.00)	^55^
13	8.90	secoisolariciresinol	[M−H]^−^	361.166 (96, 122, 165, 315)	C_20_H_26_O_6_ (− 2.77)	GNPS libraries
14	8.96	prunin	[M−H]^−^	433.114 (151, 271, 387)	C_21_H_22_O_10_ (2.31)	^56^
15	9.00	1-*O*-caffeoyl-6-*O*-(*S*)-brevifolincarboxyl-β-D-glucopyranose	[M−H]^−^	615.097 (169, 313, 465)	C_28_H_24_O_16_ (− 3.25)	GNPS libraries
16	9.10	1-*O*-(*E*)-cinnamoyl-4-galloyl-β-D-glucopyranose	[M−H]^−^	461.108 (169, 313)	C_22_H_22_O_11_ (0.00)	GNPS libraries
17	9.13	ellagic acid	[M−H]^−^	300.999 (117, 151, 173, 229, 283)	C_14_H_6_O_8_ (3.32)	^48^
18	9.68	oleuropein	[M−H]^−^	539.175 (89, 149, 275, 307, 377)	C_25_H_32_O_13_ (− 1.85)	GNPS libraries
19	9.83	phloridizin	[M−H]^−^	435.129 (167, 273)	C_21_H_24_O_10_ (0.00)	^52^
20	10.32	naringenin	[M−H]^−^	271.061 (65, 119, 151, 187)	C_15_H_12_O_5_ (0.00)	^51^
21	14.81	gingerglycolipid A	[M+HCOO]^−^	721.364 (89, 277, 397)	C_33_H_56_O_14_ (− 1.39)	^57^

Five optimized models, using the XGBoost algorithm and five different fingerprints, were concurrently utilized to screen potential XO inhibitors from the chemical constituents of the ethyl acetate fractions of the two *Balanophora* spp. by predicting the biological activity of each compound. The purpose of this ensemble process was to minimize the effect of model bias and increase overall prediction accuracy. The candidates suggested by a majority of models were considered promising compounds to explain the inhibition of XO by the ethyl acetate fractions of the *Balanophora* extracts.

### Molecular docking

Docking studies were performed using AutoDock4^58^ to predict the binding interactions between the ligands and the active site of the target protein (PDB ID: 1VDV^59^). Protein and ligand structures were prepared using ChimeraX^60^ and AutodockTools^58^, ensuring proper protonation states at pH 7.4. Protein preparation involved the removal of water molecules and non-standard residues to streamline the docking process. However, critical residues such as MTE (phosphonic acidmono-(2-amino-5,6-dimercapto-4-oxo-3,7,8a,9,10,10a-hexahydro-4H-8-oxa-1,3,9,10-tetraaza-anthracen-7-ylmethyl) ester), FAD (flavin-adenine dinucleotide), and FES (fe2/s2 (inorganic) cluster) were retained in the protein structure. MTE and FAD are essential cofactors, while FES serves as an electron carrier, all of which play indispensable roles in the catalytic mechanism of XO^61^. Their inclusion was deemed crucial to accurately represent the physiological environment and the enzymatic activity of the target protein. The docking grid box was centered at the catalytic site (x = 65.378, y = − 4.343, z = 43.596) with dimensions of 40 × 40 × 40 d and a grid spacing of 0.750 Å. Docking simulations were performed with rigorous parameters, including 100 independent genetic algorithm runs, a population size of 150, a maximum of 25 million energy evaluations, and a cap of 27,000 generations per run. Scoring was based on the lowest binding free energy (kcal/mol), and the results were validated by re-docking the orginal ligand with a root-mean-square deviation (RMSD) value of 0.87 Å, confirming the reliability of the method. Binding interactions were visualized and analyzed using PyMOL (License/Invoice No. inv56506), and BIOVIA Discovery Studio (Version 2021, San Diego: Dassault Systèmes, 2021) to identify hydrogen bonds and hydrophobic contacts.

## Results and discussion

### Comprehensive chemical constituent profiling of the ethyl acetate fractions of two *Balanophora spp.*

**Fig. 2:**
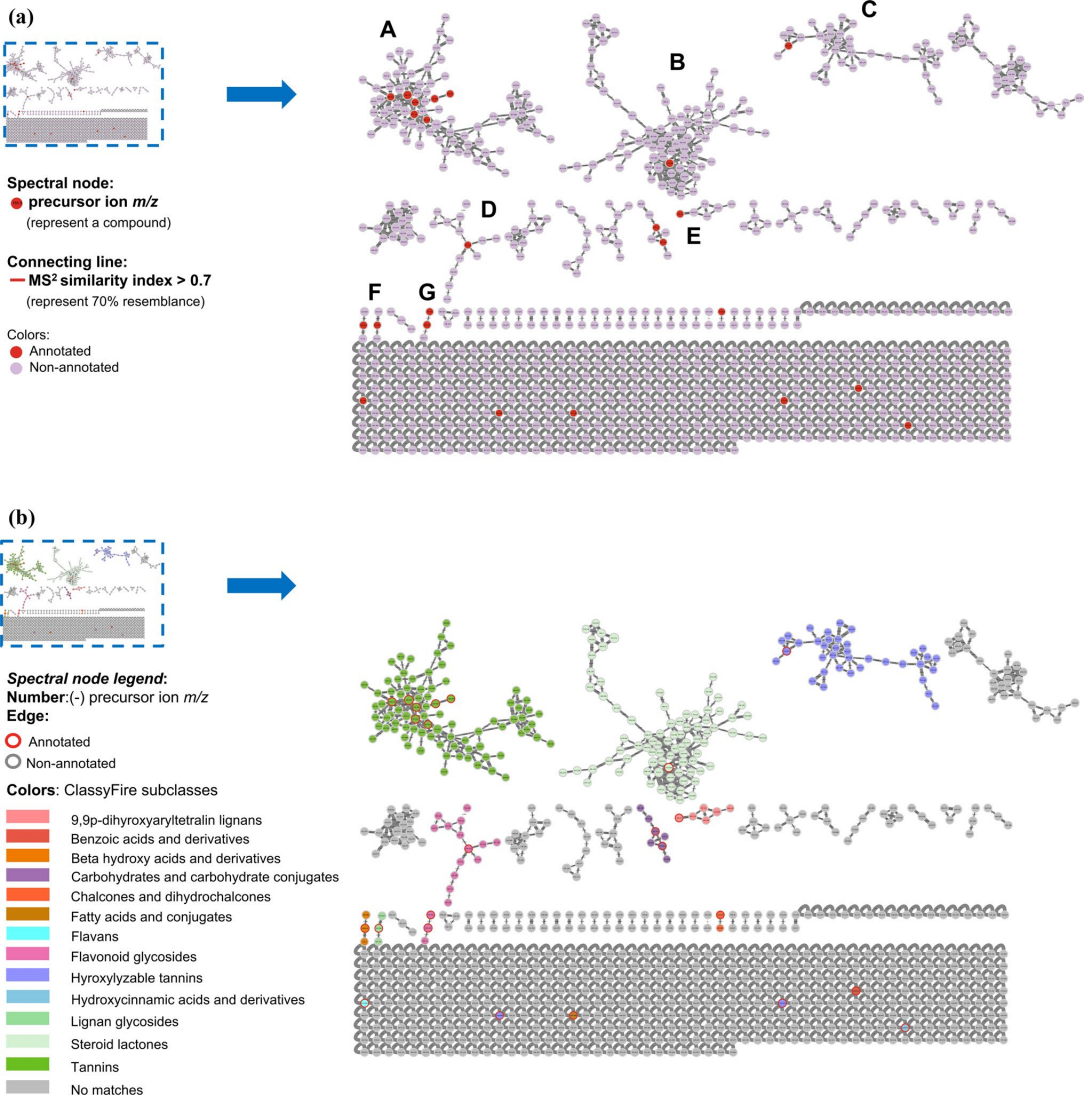

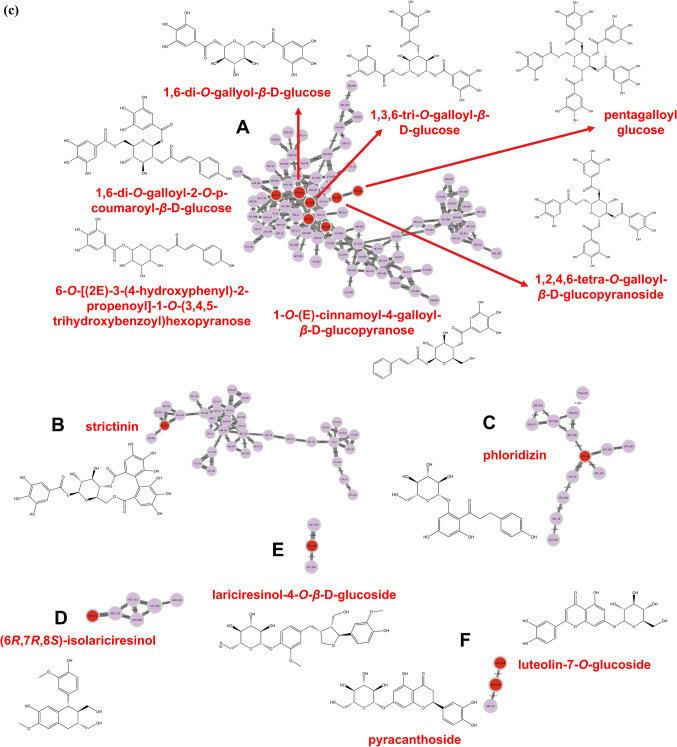
Molecular networks of the ethyl acteate extract of *B. subcupularis* (**a**); putative chemical classes of major molecular families (**b**); and putative annotations of significant representatives (**c**).

**Fig. 3:**
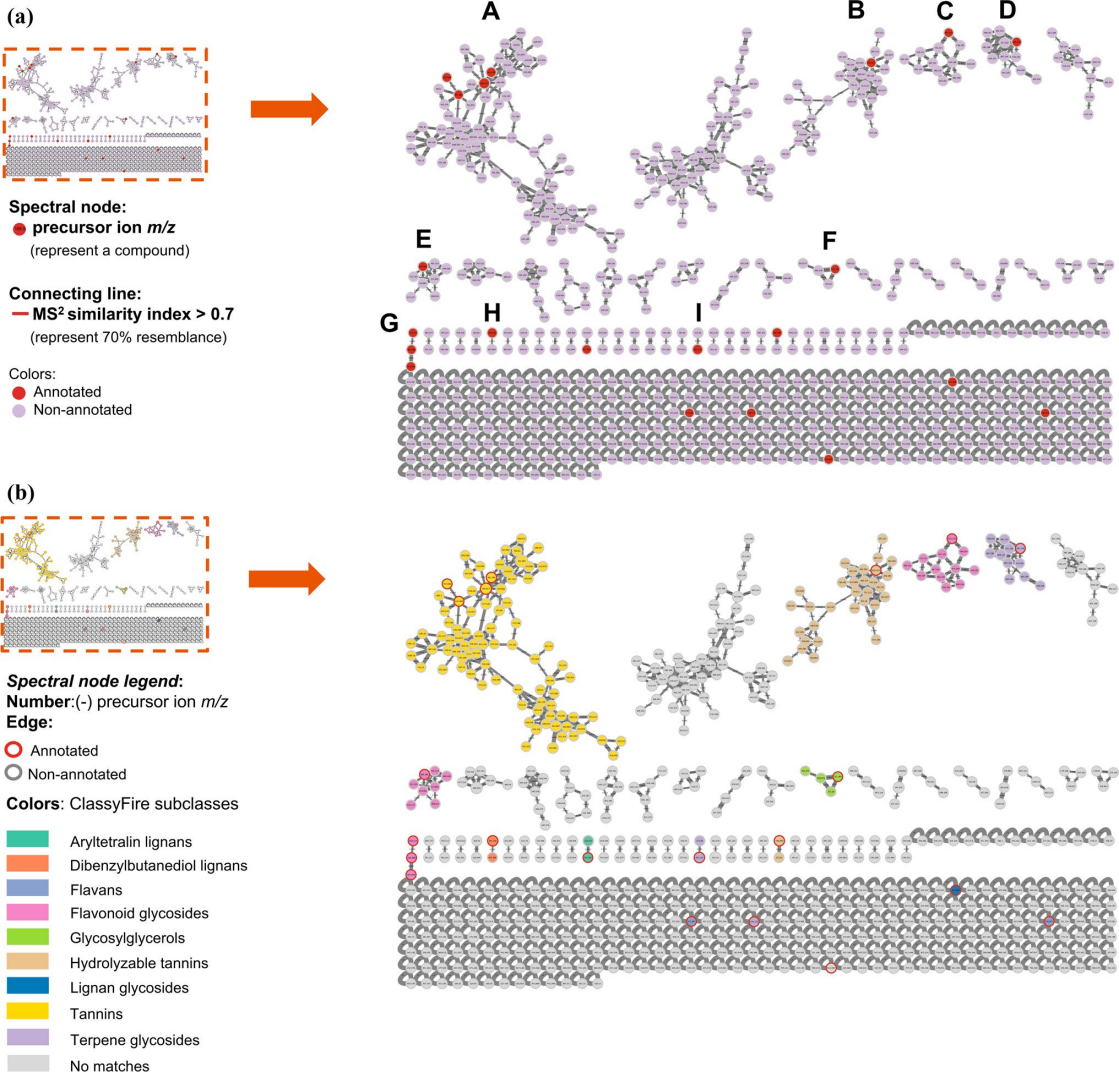

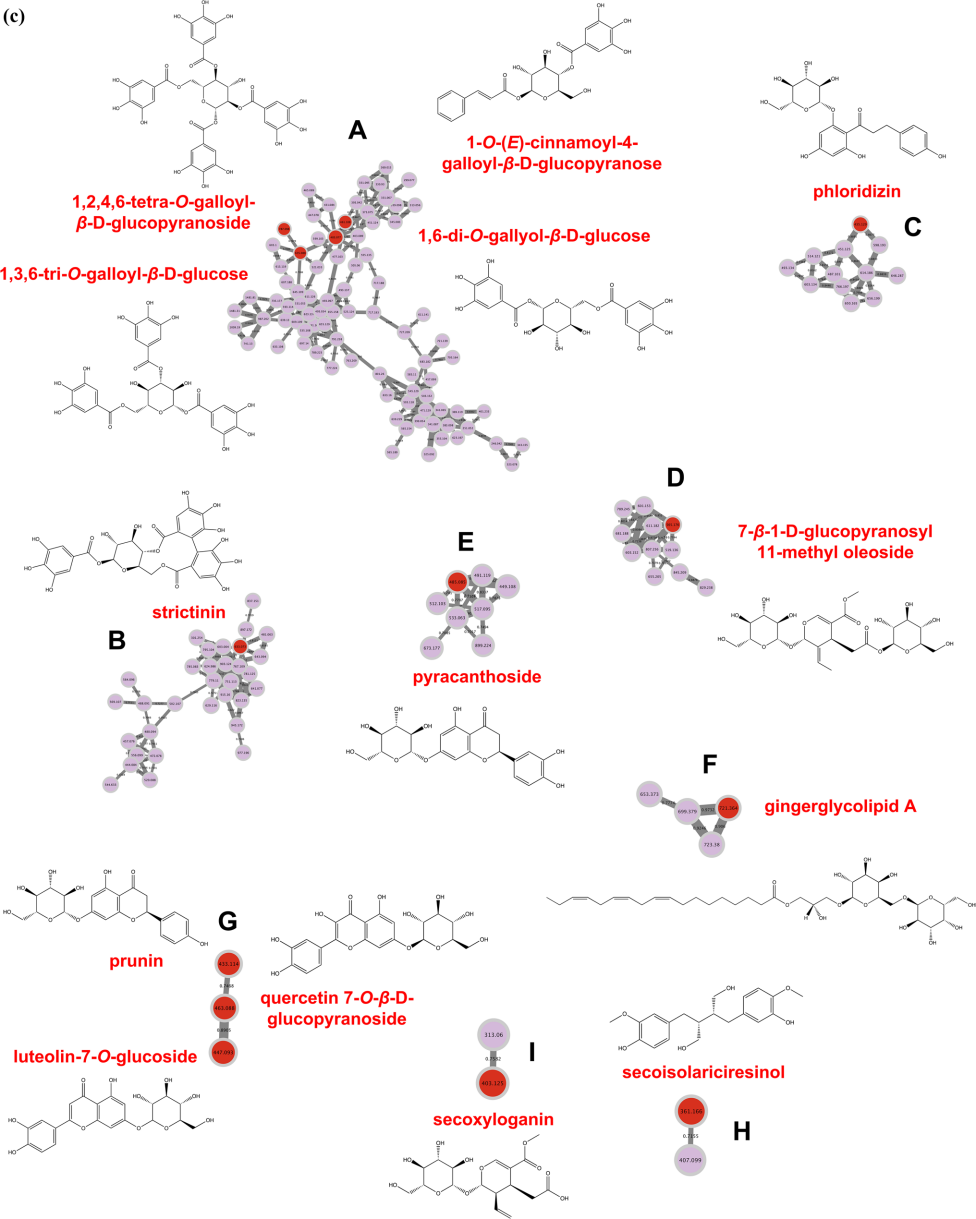
Molecular networks of the ethyl acetate extract of *B. tobiracola* (**a**); putative chemical classes of major molecular families (**b**); and putative annotations of significant representatives (**c**).

The molecular networks generated via GNPS and visualized through the software Cytoscape elucidated numerous clusters comprising annotated chemical entities. These networks were stratified based on component annotations within each cluster, as delineated in Figs. 2 and 3. In particular, Figs. 2a and b showcase the molecular network derived from negative ionization data of the *B. subcupularis* sample extract, encompassing 880 nodes and 52 molecular families. The classification of these molecular families is visually represented by node coloration in Fig. 2b. Using spectral library matching and *in silico* structure prediction tools, the chemical classes of key molecular families were tentatively determined. Similarly, the molecular network for the *B. tobiracola* sample, also based on negative ionization data, comprised 642 nodes and 56 molecular families (Fig. 3a). Prominent metabolites were organized into major molecular families, with representative compounds highlighted in Fig. 3b. These results demonstrate that molecular networking offers an effective platform for uncovering the metabolic diversity within the analyzed metabolomes, facilitating the visualization of shared and distinct metabolite classes across the studied herbal samples. By integrating the NAP tool with Reaxys data and corroborative literature sources, 23 and 21 chemical compounds were successfully annotated and identified in the *B. subcupularis* and *B. tobiracola* samples, respectively, as summarized in Tables 1 and 2.

The combination of LC-QToF-HRMS and the GNPS allowed the identification of 23 and 21 compounds from ethyl acetate fractions of *B. subcupularis* and *B. tobiracola*, respectively. Among the identified compounds, some could be identified in both samples such as strictinin; 1,6-di-*O*-galloyl-*β*-D-glucose; 1,3,6-tri-*O*-galloyl-*β*-D-glucose; 1,2,4,6-tetra-*O*-galloyl-*β*-D-glucopyranoside; pyracanthoside; luteolin-7-*O*-glucoside; ellagic acid; naringenin; and phloridizin. Out of these, strictinin and pyracanthoside were identified in genus *Balanophora* for the first time while three hydrolyzable tannins had been isolated from some other species of the genus. Ellagic acid had been isolated from *B. simaoensis* (syn. *B. fungosa* subsp. *indica*)^62^, while naringenin had been found in *B. involucrata*^63^ previously. Some other components were also identified for the first time in the genus such as 1-*O*-vanilloyl-β-D-glucose; 6-*O*-[(2*E*)-3-(4-hydroxyphenyl)-2-propenoyl]-1-*O*-(3,4,5-trihydroxybenzoyl)hexopyranose; rosmarinic acid; azelaic acid; carenone in the extract of *B. subcupularis* and secoxyloganin, taxifolin, oleuropein, and gingerlycolipid in the extract of *B. tobiracola*. Besides, some other compounds had been isolated from different species of the genus *Balanophora*. It was found that some hydrolyzable tannins structured from units of cinnamoyl-, galloyl-, caffeoyl-, brevifolincarboxyl-, and lignans (secoisolariciresinol) were characteristic of the extracts.

### Machine learning models and virtual screening

#### Machine learning model and performance assessment results

**Fig. 4:**
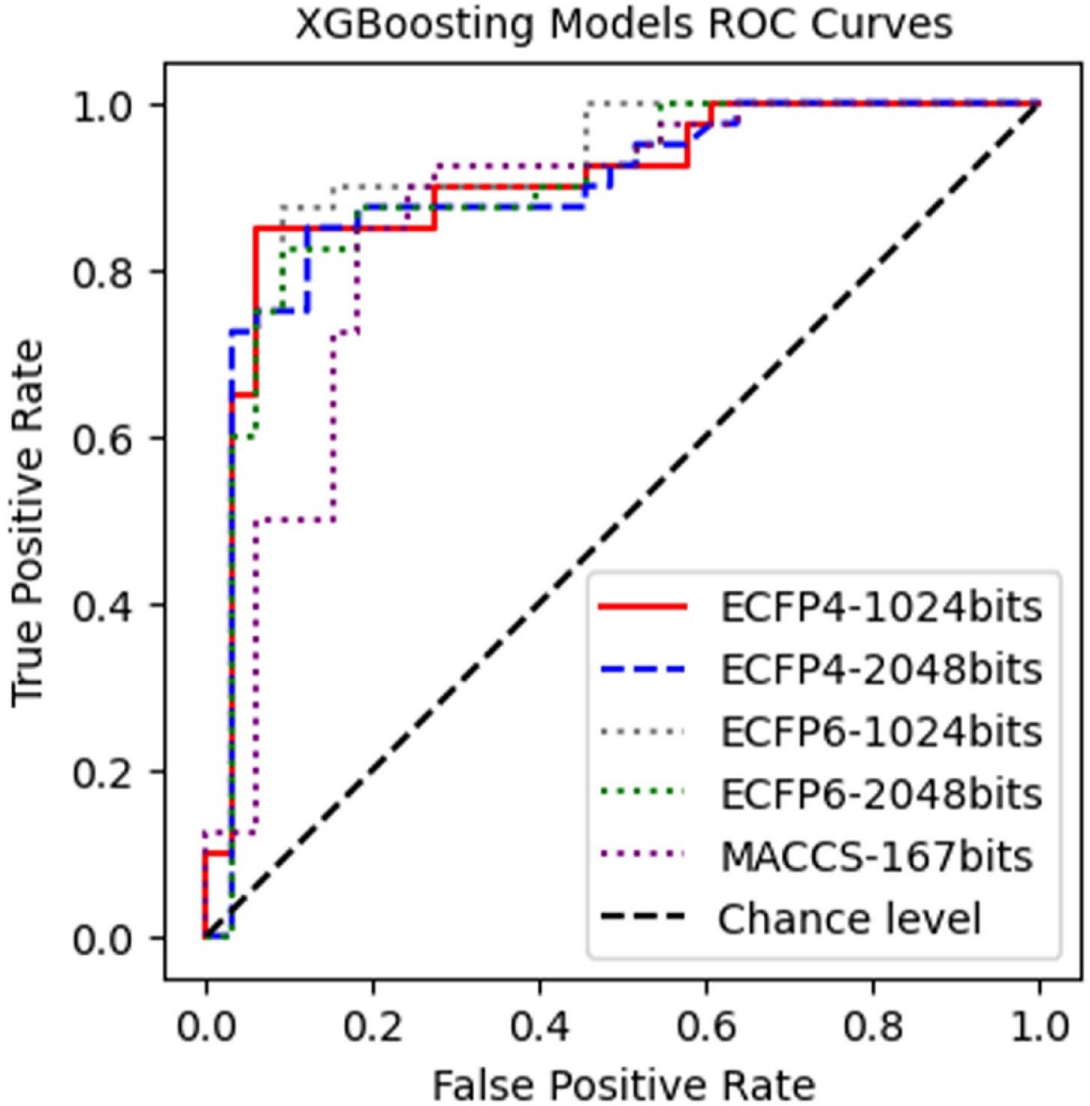
Receiver operating characteristic (ROC) curves of the models using the XGBoost algorithm.

Following optimizing the parameters along with the given conditions, the optimal hyperparameters for each model are listed in Table S2 (Supplementary Information). Other hyperparameters of the model not mentioned are kept at their default values. The results of the evaluation of the models on the test set are presented in Table 3 and Fig. 4.

**Table 3:** Performance metrics of the optimized models.

Fingerprint	tenfold-cross-validation (%)	Test set accuracy (%)	Test set F1-Score (%)	Test set AUC (%)	Test set Precision (%)	Test set Recall (%)
MACCS-167 bits	80.0	82.2	80.0	85.9	78.8	81.3
ECFP4-1024 bits	84.1	87.7	87.0	90.2	90.9	83.3
ECFP4-2048 bits	83.7	84.9	84.1	89.3	87.9	80.6
ECFP6-1024 bits	84.9	89.0	88.2	91.2	90.9	85.7
ECFP6-2048 bits	84.9	86.3	85.7	89.5	90.9	81.1

Table 3 demonstrates the performance of the XGBoost models when using MACCS, ECFP4 and ECFP6 molecular fingerprints. The MACCS-167 fingerprint model showed lower performance with 80.0% tenfold cross-validation accuracy and 82.2% test set accuracy and AUC of 85.9% and F1-score of 80.0% and precision of 78.8% and recall of 81.3%. The ECFP6-1024 fingerprint model demonstrated the highest predictive capability among all models by achieving 84.9% tenfold cross-validation accuracy and 89.0% test set accuracy along with AUC at 91.2% and precision at 90.9% and recall at 85.7% and F1-score at 88.2%. The ECFP4-1024 model demonstrated good performance through its tenfold cross-validation accuracy of 84.1% and test set accuracy of 87.7% and AUC of 90.2% and precision of 90.9% and recall of 83.3% and F1-score of 87.0%. The results demonstrated that ECFP6-1024 and ECFP4-1024 fingerprints may provide better molecular feature characterization than the MACCS-167 fingerprint, which leads to improved XGBoost-based classification results.

As shown in Fig. 4 and demonstrated by the AUC values in Table 3, all the models mentioned had AUC values greater than 85%, indicating good classification ability. The models using ECFP4 and ECFP6 fingerprints had AUC values ranging from 89.3% to 91.2%, while the model using MACCS fingerprint exhibited the lowest AUC value (85.9%), documenting poorer classification performance compared to the other models. MACCS performed worse due to its fixed bit size (167 bits), leading to a loss of detailed molecular information^64^. Additionally, MACCS only detects the presence of common functional groups without considering substructures and atomic environments like ECFP4/ECFP6, making it less accurate in distinguishing compounds^65^. It is worth noting that when increasing the fingerprint length from 1024 to 2048 bits for both ECFP4 and ECFP6 fingerprints, the prediction performance of the models decreased. This may suggest that, for the given dataset addressing the focus on XO, increasing the dimensionality to 2048 bits could lead to overfitting or noise in the model, without providing significant benefits. Table 4 shows that the optimized models had consistent classification performance in the training, validation, and test sets, suggesting good confidence in the predictive outcomes for the subsequent virtual screening process.

**Table 4:** Evaluation of model stability and overfitting prevention.

Fingerprint	Difference in accuracy values		
	Training accuracy (%)	Validation accuracy (%)	Test accuracy (%)
MACCS-167bits	85.8	83.6	82.2
ECFP4-1024bits	91.1	91.1	87.7
ECFP4-2048bits	86.9	85.5	84.9
ECFP6-1024bits	92.5	93.9	89.0
ECFP6-2048bits	90.5	90.5	86.3

The results of evaluating the overfitting and stability of the models are shown through the difference in model accuracy when calculated on different data sets including training set, evaluation set, and test set in Table 4.

The data in Table 4 show that the optimized models had differences of less than 5% between the training set and the test and validation sets, indicating that the models are not overfitting. Furthermore, the discrepancies between the test and validation sets after hyperparameter tuning were less than 1.5%, confirming the consistency of the models. Consequently, the analysis suggests that models utilizing ECFP4, ECFP6, and MACCS fingerprints exhibit stable classification, avoiding overfitting, and instilling high confidence in the predictive outcomes for the subsequent virtual screening process.

To further evaluate the robustness of the proposed ML pipeline, an external validation step was performed using two independent enzyme inhibitor datasets including an HDAC2 dataset^66^ and a newly curated HDAC3 dataset. These datasets are chemically and biologically distinct from the primary XO dataset and were used to test model performance without retraining. The external validation results confirmed that the pipeline maintains high predictive performance across different targets, with AUC values ranging from approximately 0.84 to 0.92 (Table S3 – Supplementary Information). Differences observed in Precision and Recall between the two datasets reflect their respective class distributions, further emphasizing the importance of using multiple evaluation metrics for robust assessment. Detailed experimental setup and complete performance metrics are provided in Tables S3-S4 of the Supplementary Information.

#### Virtual screening results

A dataset consisting of 33 structures identified in the ethyl acetate extracts of the two *Balanophora* species was used to search for potential compounds that inhibit XO. Five models were adduced to assess the activity of each structure. During the screening process, 20 compounds were randomly selected from the training dataset, all labeled as active by at least one model, to serve as decoy compounds. The screening results are summarized in Table 5. The screening results showed that all four models were able to detect all the decoy compounds, demonstrating the capability of the models to search for active compounds. To prioritize selecting structures with genuine activity, the current study focuses on choosing the group of structures that satisfy the most models.

**Table 5:** Predicted active compounds by the five models using XGBoost algorithm.

Fingerprint	Decoy compounds found	Predicted active compounds
MACCS-167bits	20/20	1-(3,4-dihydroxyphenyl)-6,7-dihydroxy-1,2-dihydro-2,3-naphthalenedicarboxylic acid taxifolin
ECFP4-1024bits	20/20	1-*O*-caffeoyl-6-*O*-(*S*)-brevifolincarboxyl-β-D-glucopyranose taxifolin
ECFP4-2048bits	20/20	1-*O*-caffeoyl-6-*O*-(*S*)-brevifolincarboxyl-β-D-glucopyranose taxifolin
ECFP6-1024bits	20/20	none
ECFP6-2048bits	20/20	1-*O*-caffeoyl-6-*O*-(*S*)-brevifolincarboxyl-β-D-glucopyranose taxifolin

From Table 5, it can be seen that taxifolin and 1-*O*-caffeoyl-6-*O*-(*S*)-brevifolincarboxyl-β-D-glucopyranose were predicted by four and three models to be active, respectively, and 1-(3,4-dihydroxyphenyl)-6,7-dihydroxy-1,2-dihydro-2,3-naphthalenedicarboxylic acid was found to be active by only one model, which was XGB-MACCS. All these compounds also fall within the application domain, which means that the established models could give promising predictions on these compounds.

Strikingly, all three identified compounds were exclusively found in the ethyl acetate fraction of *B. tobiracola*, with none detected in *B. subcupularis*. Moreover, the ethyl acetate fraction of *B. tobiracola* exhibited significantly stronger XO inhibitory activity compared to that of *B. subcupularis*, as evidenced by its markedly lower IC₅₀ value (11.87 ± 1.28 µg/mL vs. 48.41 ± 1.56 µg/mL)^14^. This substantial difference suggests that *B. tobiracola* might harbor more potent XO-inhibitory constituents. Among the identified compounds, 1-(3,4-dihydroxyphenyl)-6,7-dihydroxy-1,2-dihydro-2,3-naphthalenedicarboxylic acid, taxifolin, and 1-*O*-caffeoyl-6-*O*-(*S*)-brevifolincarboxyl-β-D-glucopyranose were predicted to be the principal contributors to this superior inhibitory effect. These findings highlight the potential of *B. tobiracola* as a more promising source of natural XO inhibitors compared to *B. subcupularis*.

#### Docking results

To validate the accuracy and reliability of our elaborated molecular docking protocol, a re-docking procedure was performed using crystal structures of XO complexed with known inhibitors. The docking methodology was assessed by comparing the binding conformations of the re-docked ligands with their experimentally determined crystal poses, evaluating the RMSD values. Additionally, the correlation between docking scores and Gibbs free energy of binding (delta G) calculated from reported inhibition constants (K_i_) was analyzed to ensure predictive robustness. The results of the re-docking validation are summarized in Table S5—Supplementary Information.

The re-docking validation confirmed that the molecular docking protocol used in this study is reliable for evaluating XO inhibitors. The low RMSD values across all complexes (RMSD < 2 Å) demonstrated that the docking method effectively reproduces the experimentally determined ligand-binding conformations, ensuring accuracy in the subsequent screening processes. Moreover, the strong correlation between docking scores and experimental binding affinities (R^2^ = 0.95) suggested that the computational predictions align well with experimental inhibitory effects.

**Fig. 5:**
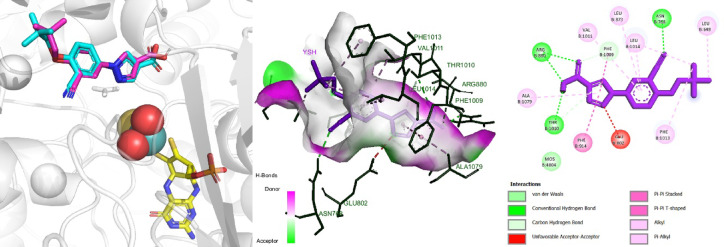
The interaction between the ligand and the enzyme is re-established in the 1VDV complex; *Protein (grey cartoon), MOS (sphere), MTE (yellow), crystal ligand (blue), redock ligand (magenta)*.

Among the tested XO crystal structures, 1VDV emerged as the most representative model, with a low RMSD, strong binding affinity, and essentials cofactors within the active site^59^. The presence of key interactions with critical residues such as Asn768, Glu802, Arg880, Phe914, and Thr1010 further reinforced its relevance for assessing potential inhibitors (Fig. 5).

Overall, the validated docking approach provided a robust framework for identifying novel XO inhibitors, ensuring that the predicted binding affinities and interactions are biologically meaningful. These findings lay a solid foundation for the subsequent virtual screening in the discovery of potent natural XO inhibitors from *Balanophora* species.

**Fig. 6:**
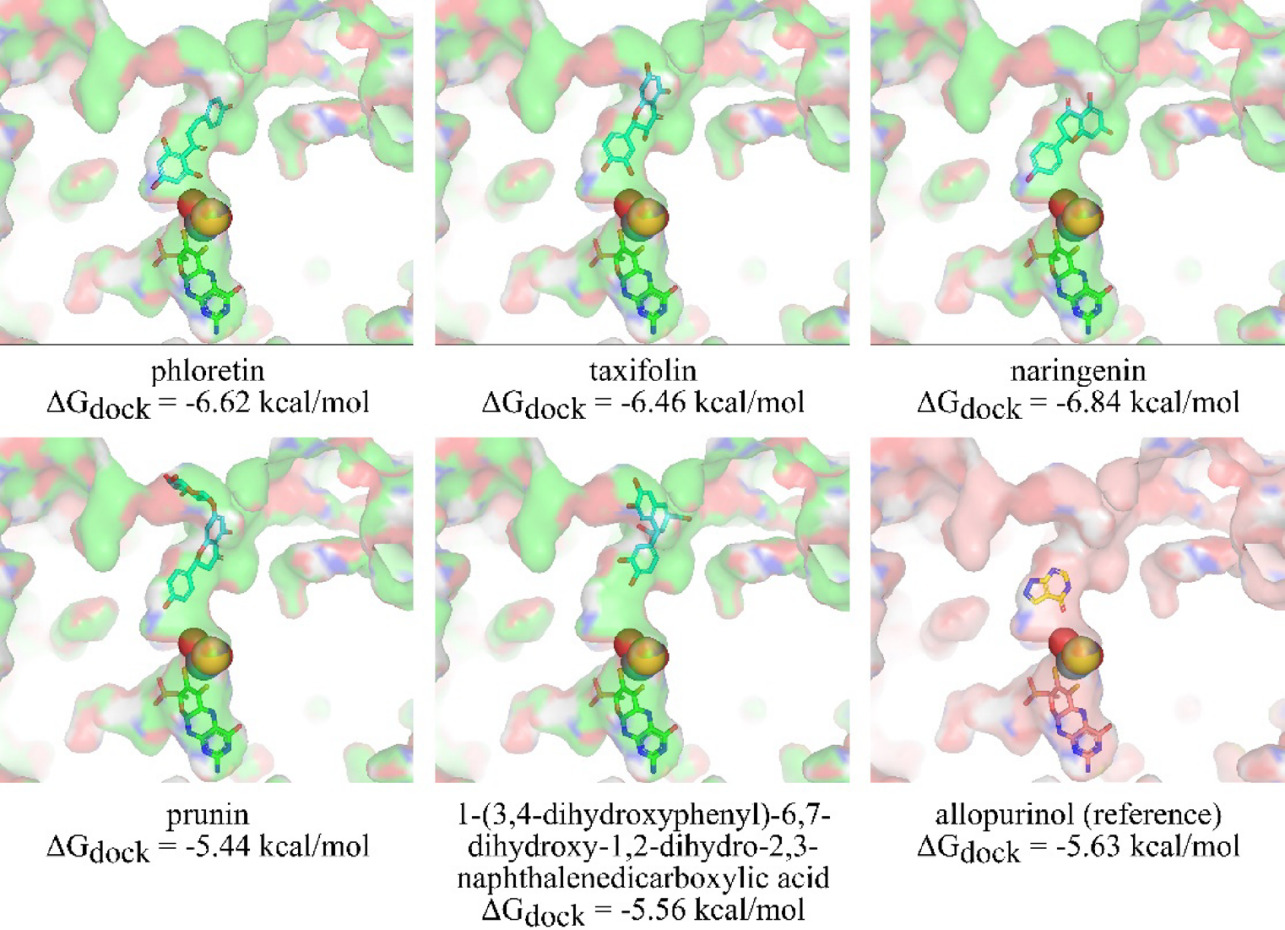
Result of docking studies for discovering potential XO inhibitor in two *Balanophora* species fractions.

Utilizing the validated docking protocol, we screened all identified compounds from extracts of *B. subcupularis* and *B. tobiracola* to assess their potential as XO inhibitors (Table S6, Supplementary Information). The docking results revealed that five compounds demonstrated binding affinities equal to or better than allopurinol, a clinically approved XO inhibitor (Fig. 6).

Notably, among these compounds, several have been previously reported to exhibit hypouricemic effects or XO inhibitory activity, further supporting their relevance in gout and hyperuricemia treatment. The top-performing compounds included naringenin (− 6.84 kcal/mol), phloretin (-6.62 kcal/mol), and taxifolin (-6.46 kcal/mol), all of which exhibited stronger docking scores compared to allopurinol (-5.63 kcal/mol). Naringenin and taxifolin are well-documented flavonoids known for their uric acid-lowering effects^67^,^68^. Previous studies highlighted that taxifolin can inhibit XO and reduce uric acid levels *in vivo*^67^, while naringenin was also reported to exhibit hypouricemic effects at a high dose (100 mg/kg)^69^. Phloretin was studied for its effects on XO, showing comparable inhibitory activity to allopurinol^70^. Besides the three above-mentiond phenolic compounds, 1-(3,4-dihydroxyphenyl)-6,7-dihydroxy-1,2-dihydro-2,3-naphthalenedicarboxylic acid (-5.56 kcal/mol) and prunin (-5.44 kcal/mol) exhibited comparable binding affinities, thus suggesting a promising role in XO inhibition, too.

**Fig. 7:**
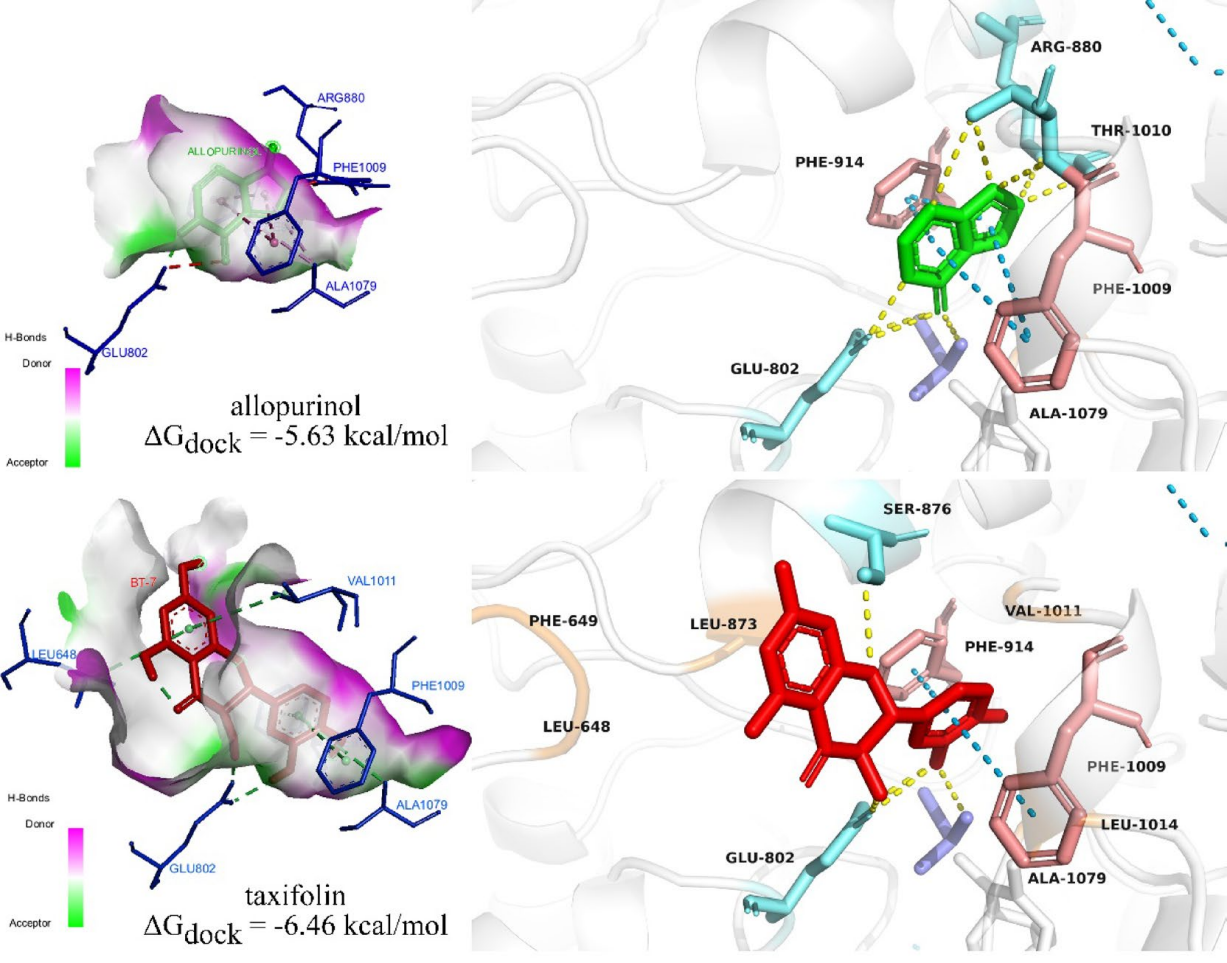
Interactions of taxifolin (top) and allopurinol (bottom) with the active site of XO.

Taxifolin emerged as a key contributor due to its favorable docking score, previously reported hypouricemic effects, and identification as an XO inhibitor through both ML-based screening and docking analysis. Figure 7 illustrates the detailed interactions of taxifolin with the active site of XO. The interaction with allopurinol is shown for comparison.

The docking analysis suggested that taxifolin interacts with XO through a network comparable to that of the established urate-lowering drug allopurinol. Recently, Pan et al.^61^ pointed out that the following amino acids residues were the most important for allopurinol binding, namely Arg880, Ala1079, and Thr1010. These three residues made at least one hydrogen bonds with allopurinol. The results from our finding support that claim and also allopurinol can establish another hydrogen bonds with Glu802. Similarly, taxifolin also formed hydrogen bonds with 3/4 key amino acids in the active site as allopurinol. Though lack of hydrogen bonds between the ligand and Thr1010, taxifolin and XO have additionalyl two strong to mild hydrogen bonds at residue Ser876 with the distance range from 2.22 to 3.23 Å. Besides, several reports showed that the amino acids Glu802 and Arg880 were critical in the hydroxylation of substrate xanthine^71^.

Both compounds establish key π-π stacking interactions with Phe914 and Phe1009, key residues contributing to ligand stabilization within the active site^72^. In comparison to allopurinol, taxifolin formed multiple hydrophobic interactions, including Leu648 and Phe649, Leu873, Val1011, and Leu1014. This can be explained as taxifolin contains more aromatic rings in contrast to allopurinol structure. Additionally, hydrophobic interaction between taxifolin and Leu648 can lead to the stabilization of the compound inside the active site^73^. The distance between taxifolin and the molybdenum cofactor (4.7 Å) closely resembles that of allopurinol (4.9 Å), indicating that taxifolin can penetrate the catalytic core of XO to a similar extent. From the findings of the molecular docking in this research in comparison with previous research, taxifolin can be considered a potent XO inhibitor.

Allopurinol, known as the common treatment for hyperuricemia and gout, is associated with hypersensitivity reactions (HSRs) that can range from mild skin rashes to severe, life-threatening conditions such as Stevens-Johnson syndrome (SJS) and toxic epidermal necrolysis (TEN)^74,75^. The purine-like structure of allopurinol may interfere with other purine metabolic pathways, potentially resulting in such adverse effects. Additionally, both allopurinol and its metabolite, oxypurinol, can bind to specific human leukocyte antigen (HLA) molecules, notably HLA-B*58:01, facilitating the presentation of the drug as an antigen to T cells and triggering immune responses^76,77^.

Taxifolin, in contrast, is a naturally occurring flavonoid without a purine core, potentially reducing the risk of such adverse effects. Moreover, taxifolin has been extensively studied for its diverse pharmacological properties, including antioxidant activity^78^, anti-inflammatory effects^79^, cardiovascular protection^80^, potential anticancer properties^81^, among others. Given its strong XO inhibitory potential, supposedly lower risk of purine-related side effects, and additional health benefits, taxifolin emerges as a promising natural lead compound for developing next-generation XO inhibitors. The potential of taxifolin as an XO inhibitor was finally unveiled by ML-based virtual screening and docking experiments in the context of investigating extracts of *Balanophor spp.* Consistent with prior *in vitro* evidence, the ethyl acetate fraction of *B. tobiracola* consistently outperformed that of *B. subcupularis* in XO inhibition, which is consistent with the localization of predicted actives in these fractions. This integrative view links model-derived hypotheses with observed bioactivity and clarifies pharmacological relevance. In this framework, the computational results complement experimental evidence and help prioritize compounds for subsequent validation.

## Conclusion

This study successfully identified constituents from the ethyl acetate extracts of *Balanophora subcupularis* and *Balanophora tobiracola* using LC-QToF-HRMS analysis, providing a comprehensive phytochemical profile of these medicinal plants. Several compounds were identified for the first time in the *Balanophora* genus, including strictinin and pyracanthoside, expanding the known chemical diversity of this plant family. To further elucidate the xanthine oxidase (XO) inhibitory potential of these extracts, machine learning (ML)-based virtual screening models were developed using a diverse dataset of 483 known XO inhibitors. The ML models demonstrated high predictive accuracy, enabling the efficient selection of promising candidate compounds in the extracts of the two *Balanophora spp.* However, the elaborated procedure might also be applied to further extracts of other medicinal plants. By integrating ML screening with molecular docking simulations, this study proposed taxifolin and 1-(3,4-dihydroxyphenyl)-6,7-dihydroxy-1,2-dihydro-2,3-naphthalenedicarboxylic acid as key contributors to the stronger inhibitory effect observed in *B. tobiracola* compared to *B. subcupularis*. Taxifolin emerged as the most promising XO inhibitor, being reported for the first time in *B. tobiracola*. It was predicted as active by four out of five ML models, and exhibiting strong docking interactions mimicking allopurinol. The developed models proved suitability for the search of novel XO inhibitors in extracts of pharmaceutical species. Taken together, our ML and docking guided results, supported by fraction-level activity, should be regarded as hypothesis-generating pending compound-level validation with authentic standards and targeted XO assays. The absence of the latter is considered a limitation of the current study and must be taken into account in future work. However, the aim of this project was to gain insight based on ML-based approaches, and this goal was achieved. Future research should focus on structural optimization of the hit compound taxifolin to explore its full therapeutic potential as a safer alternative to allopurinol in hyperuricemia and gout management.

## Supplementary Information

Supplementary Material 1

## Data Availability

The datasets used and analysed during the current study available from the corresponding author Do Thi Mai Dung ( dungdtm@hup.edu.vn ) on reasonable request. The datasets generated and analysed during the current study are available at https://github.com/myLab-UET/mylab-xanthine-oxidase/tree/main .
