# Supplementary material for: Unveiling potent xanthine oxidase inhibitors in two Balanophora spp. using machine learning-based virtual screening and molecular docking approach
Source: Sci Rep. 2025 Dec 16;15:43877. doi: 10.1038/s41598-025-32282-6 (PMC12708773; doi:10.1038/s41598-025-32282-6)
Supplement: Supplementary file 1 — Supplementary Material 1 [file 41598_2025_32282_MOESM1_ESM.docx]

Supporting Information

**Unveiling Potent Xanthine Oxidase Inhibitors in two *Balanophora* spp. using Machine Learning-based Virtual Screening and Molecular Docking Approach**

*Nguyen Ngoc An^a^, Dao Quang Tung^b^, Le Van Tue^a^, Nguyen Thanh Son^a^, Nguyen Thanh Tung^c^, Huong-Giang Le^d^, Thai Chinh Tam^c^, Nguyen Thị Thuan^c^ , Daniel Baecker^e^*, Do Thi Mai Dung^c^**

^a^VNU University of Engineering and Technology, 144 Xuan Thuy, Cau Giay, Hanoi 100000, Vietnam

^b^ Stockholm University, SE-106 91 Stockholm, Sweden

^c^ Hanoi University of Pharmacy, 13 - 15 Le Thanh Tong, Hoan Kiem 11021, Hanoi 100000, Vietnam

^d^ Department of Pharmacognosy and Traditional Pharmacy, School of Pharmacy, University of Medicine and Pharmacy at Ho Chi Minh City, Ho Chi Minh City 700000, Vietnam

^e^ Department of Pharmaceutical and Medicinal Chemistry, Institute of Pharmacy, Freie Universität Berlin, Königin-Luise-Straße 2+4, 14195 Berlin, Germany

**TABLE OF CONTENTS**

| **Order** | **Name of Table** | **Page** |
| --- | --- | --- |
| 1 | Table S1. Pairwise Tc value distribution of the input dataset | SI-1 |
| 2 | Table S2. Optimized hyperparameter sets for five models using XGBoost algorithm | SI-3 |
| 3 | Table S3: Performance metrics of the optimized models on HDAC2 and HDAC3 datasets (External Validation) | SI-4 |
| 4 | Table S4: Hyperparameter configurations used for external validation | SI-6 |
| 5 | Table S5. Summary of XO Re-docking Results | SI-7 |
| 6 | Table S6: Docking of identified components in the ethyl acetate extracts of two *Balanophora* spp. | SI-9 |

**Table S1. Pairwise Tc value distribution of the input dataset**

| **Tc threshold** | **Frequency** | **Percentage (%)** |
| --- | --- | --- |
| **0.00 - 0.02** | 596 | 0.51 |
| **0.02 - 0.04** | 1157 | 0.99 |
| **0.04 - 0.06** | 3847 | 3.29 |
| **0.06 - 0.08** | 10455 | 8.94 |
| **0.08 - 0.10** | 18766 | 16.05 |
| **0.10 - 0.12** | 22214 | 19 |
| **0.12 - 0.14** | 18577 | 15.89 |
| **0.14 - 0.16** | 12080 | 10.33 |
| **0.16 - 0.18** | 8135 | 6.96 |
| **0.18 - 0.20** | 4917 | 4.21 |
| **0.20 - 0.22** | 3952 | 3.38 |
| **0.22 - 0.24** | 2354 | 2.01 |
| **0.24 - 0.26** | 1686 | 1.44 |
| **0.26 - 0.28** | 1101 | 0.94 |
| **0.28 - 0.30** | 815 | 0.7 |
| **0.30 - 0.32** | 711 | 0.61 |
| **0.32 - 0.34** | 492 | 0.42 |
| **0.34 - 0.36** | 483 | 0.41 |
| **0.36 - 0.38** | 412 | 0.35 |
| **0.38 - 0.40** | 279 | 0.24 |
| **0.40 - 0.42** | 251 | 0.21 |
| **0.42 - 0.44** | 278 | 0.24 |
| **0.44 - 0.46** | 240 | 0.21 |
| **0.46 - 0.48** | 237 | 0.2 |
| **0.48 - 0.50** | 169 | 0.14 |
| **0.50 - 0.52** | 218 | 0.19 |
| **0.52 - 0.54** | 195 | 0.17 |
| **0.54 - 0.56** | 175 | 0.15 |
| **0.56 - 0.58** | 225 | 0.19 |
| **0.58 - 0.60** | 186 | 0.16 |
| **0.60 - 0.62** | 221 | 0.19 |
| **0.62 - 0.64** | 189 | 0.16 |
| **0.64 - 0.66** | 161 | 0.14 |
| **0.66 - 0.68** | 140 | 0.12 |
| **0.68 - 0.70** | 134 | 0.11 |
| **0.70 - 0.72** | 132 | 0.11 |
| **0.72 - 0.74** | 139 | 0.12 |
| **0.74 - 0.76** | 132 | 0.11 |
| **0.76 - 0.78** | 83 | 0.07 |
| **0.78 - 0.80** | 66 | 0.06 |
| **0.80 - 0.82** | 66 | 0.06 |
| **0.82 - 0.84** | 64 | 0.05 |
| **0.84 - 0.86** | 38 | 0.03 |
| **0.86 - 0.88** | 23 | 0.02 |
| **0.88 - 0.90** | 20 | 0.02 |
| **0.90 - 0.92** | 22 | 0.02 |
| **0.92 - 0.94** | 11 | 0.01 |
| **0.94 - 0.96** | 1 | 0 |
| **0.96 - 0.98** | 15 | 0.01 |
| **0.98 - 1.00** | 26 | 0.02 |

**Table S2: Optimized hyperparameter sets for five models using XGBoost algorithm**

| **Fingerprint** | **Hyperparameter** |
| --- | --- |
| MACCS-167bits | (colsample_bytree=0.5, learning_rate=0.01, max_depth=5, min_child_weight=3, n_estimators=80, reg_lambda=0) |
| ECFP4-1024bits | (colsample_bytree=0.5, learning_rate=0.1, max_depth=2, min_child_weight=5,  n_estimators=170,  reg_lambda=0.1) |
| ECFP6-1024bits | (colsample_bytree=0.5, learning_rate =0.1, max_depth=2, min_child_weight=3, n_estimators=130, reg_lambda = 1) |
| ECFP6-2048bits | (learning_rate =0.1, max_depth=9, min_child_weight=7, n_estimators=60, reg_lambda = 1) |
| ECFP4-2048bits | (colsample_bytree=0.5, learning_rate =0.1, max_depth=2, min_child_weight=7, n_estimators=120, reg_lambda =0.1) |

**Table S3: Performance metrics of the optimized models on HDAC2 and HDAC3 datasets (External Validation)**

| **Dataset** | **Fingerprint** | **10-fold-cross-validation** | **Test set accuracy** | **Test set F1-Score** | **Test set AUC** | **Test set Precision** | **Test set Recall** |
| --- | --- | --- | --- | --- | --- | --- | --- |
| HDAC2 | MACCS-167 bits | 82.73% | 81.85% | 69.46% | 88.6% | 63.74% | 76.32% |
|  | ECFP4-1024 bits | 83.77% | 85.05% | 74.07% | 91.19% | 65.93% | 84.51% |
|  | ECFP4-2048 bits | 83.73% | 84.7% | 72.96% | 90.33% | 63.74% | 85.29% |
|  | ECFP6-1024 bits | 83.57% | 85.77% | 74.36% | 91.17% | 63.74% | 89.23% |
|  | ECFP6-2048 bits | 84.09% | 86.48% | 76.25% | 92.06% | 67.03% | 88.41% |
| HDAC3 | MACCS-167 bits | 80.39% | 80.61% | 86.78% | 85.7% | 94.59% | 80.15% |
|  | ECFP4-1024 bits | 77.82% | 75.76% | 84.5% | 83.95% | 98.19% | 74.15% |
|  | ECFP4-2048 bits | 83.18% | 83.64% | 88.51% | 89.7% | 93.69% | 83.87% |
|  | ECFP6-1024 bits | 80.61% | 81.21% | 87.24% | 86.08% | 95.5% | 80.3% |
|  | ECFP6-2048 bits | 82.32% | 81.82% | 87.29% | 90.28% | 92.79% | 82.4% |

To evaluate the robustness of the developed machine learning pipeline, an external validation was conducted using two independent enzyme inhibitor datasets: **HDAC2** and **HDAC3**. These datasets are chemically and biologically distinct from the primary XO dataset, enabling the assessment of the pipeline’s performance on external dataset.

The HDAC2 dataset consists of 899 active compounds and 1902 inactive compounds, representing a moderately imbalanced distribution with more inactive molecules. In contrast, the HDAC3 dataset contains 739 active compounds and 359 inactive compounds, resulting in an imbalance in the opposite direction, with a predominance of active compounds. This difference in class distribution is crucial, as it allows us to observe how precision and recall metrics are influenced by imbalance. Specifically, lower precision for HDAC2 and higher precision for HDAC3 are consistent with their respective proportions of actives and inactives.

The ML pipeline developed on the XO dataset was reused to process and evaluate the external datasets. Specifically, molecular representations were re-encoded using MACCS, ECFP4, and ECFP6 fingerprints, followed by hyperparameter optimization and model training to assess whether the established pipeline remained effective on HDAC2 and HDAC3. This approach ensured methodological consistency between the internal and external analyses and allowed us to directly evaluate the adaptability and robustness of the pipeline. Performance was evaluated using the same.

For both HDAC2 and HDAC3, the pipeline achieved consistently high accuracy and AUC values, demonstrating that the modeling framework initially developed for the XO dataset can be effectively applied to external datasets. For HDAC2, AUC values ranged from 88.6% to 92.06%, with accuracy between 81.85% and 86.48%. Recall values were relatively high (76.32%–89.23%), while precision values were lower (63.74%–67.03%), reflecting the dominance of inactives in the dataset. For HDAC3, AUC values ranged from 83.95% to 90.28%, and accuracy from 75.76% to 83.64%, with higher precision values (83.95%–98.19%) and slightly lower recall (74.15%–83.87%) due to the higher proportion of actives.

**Table S4. Hyperparameter configurations used for external validation**

| **Dataset** | **Fingerprint** | **Hyperparameter** |
| --- | --- | --- |
| HDAC2 | MACCS-167bits | {'colsample_bytree': 0.7, 'learning_rate': 0.1, 'max_depth': 4, 'min_child_weight': 7, 'n_estimators': 100, 'reg_lambda': 0.01} |
|  | ECFP4-1024bits | {'colsample_bytree': 0.9, 'learning_rate': 0.1, 'max_depth': 4, 'min_child_weight': 5, 'n_estimators': 100, 'reg_lambda': 1} |
|  | ECFP6-1024bits | (colsample_bytree=0.5, learning_rate =0.1, max_depth=2, min_child_weight=3, n_estimators=130, reg_lambda = 1) |
|  | ECFP6-2048bits | {'colsample_bytree': 0.7, 'learning_rate': 0.1, 'max_depth': 4, 'min_child_weight': 3, 'n_estimators': 100, 'reg_lambda': 0.01} |
|  | ECFP4-2048bits | {'colsample_bytree': 0.7, 'learning_rate': 0.1, 'max_depth': 4, 'min_child_weight': 3, 'n_estimators': 100, 'reg_lambda': 0.1} |
| HDAC3 | MACCS-167bits | {'colsample_bytree': 0.7, 'learning_rate': 0.1, 'max_depth': 3, 'min_child_weight': 5, 'n_estimators': 60, 'reg_lambda': 1} |
|  | ECFP4-1024bits | {'colsample_bytree': 0.5, 'learning_rate': 0.1, 'max_depth': 2, 'min_child_weight': 3, 'n_estimators': 30, 'reg_lambda': 0} |
|  | ECFP6-1024bits | {'colsample_bytree': None, 'learning_rate': 0.1, 'max_depth': 2, 'min_child_weight': 7, 'n_estimators': 60, 'reg_lambda': 0.1} |
|  | ECFP6-2048bits | {'colsample_bytree': None, 'learning_rate': 0.1, 'max_depth': 3, 'min_child_weight': 5, 'n_estimators': 50, 'reg_lambda': 1} |
|  | ECFP4-2048bits | {'colsample_bytree': None, 'learning_rate': 0.1, 'max_depth': 3, 'min_child_weight': 5, 'n_estimators': 50, 'reg_lambda': 0} |

**Table S5. Summary of XO Re-docking Results**

| **Complex [Reference]** | **K_i_ (nM)** | **Delta G (kcal/mol)** | **Docking score (kcal/mol)** | **RMSD redock (Å)** | **Redock pose** |
| --- | --- | --- | --- | --- | --- |
| 3UNA [59] | 10^8^ | -1.42 | -4.08 | 0.71 | 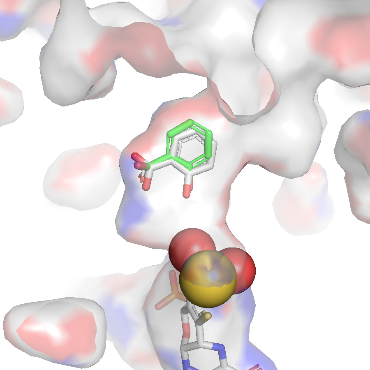 |
| 1N5X [60] | 900 | -8.57 | -6.70 | 0.70 | 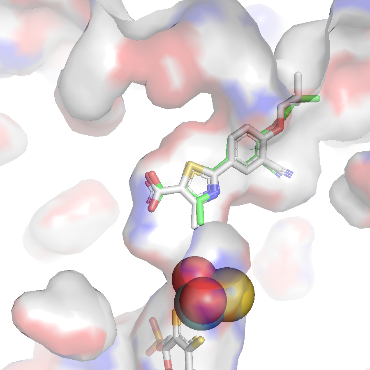 |
| 1VDV [26] | 16 | -11.06 | -7.26 | 0.87 | 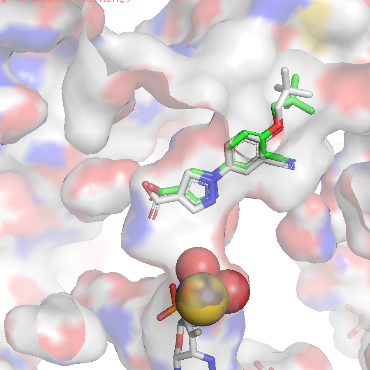 |
| 3AM9 [61] | 5.7 | -11.69 | -7.26 | 0.16 | 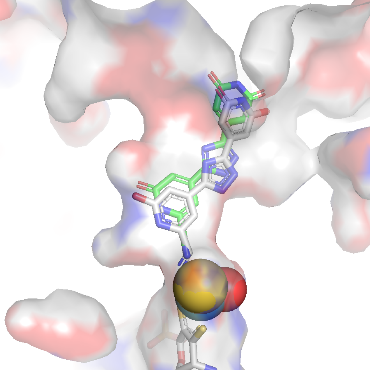 |
| 3NVY [62] | 1200 | -8.40 | -6.27 | 1.30 | 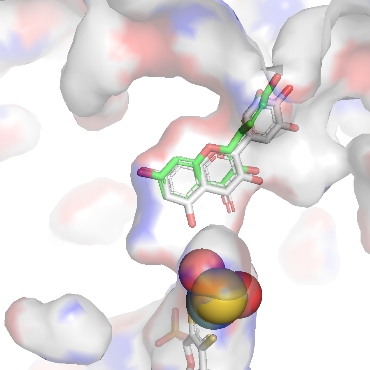 |
| 3NVZ [63] | 95000 | -5.70 | -4.87 | 1.45 | 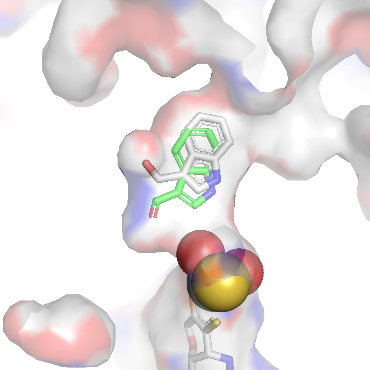 |
|  |  | **R^2^ = 0.95** | |  |  |

**Table S6: Docking of identified components in the ethyl acetate extracts of two *Balanophora* spp.**

| **Compound** | **SMILES** | **Docking score (kcal/mol)** | **Dock pose** |
| --- | --- | --- | --- |
| BS-1 | OC[C@@H](O1)[C@@H](O)[C@H](O)[C@]1(CO)O[C@@H](O2)[C@H](O)[C@@H](O)[C@H](O)[C@H]2CO | -0.19 | 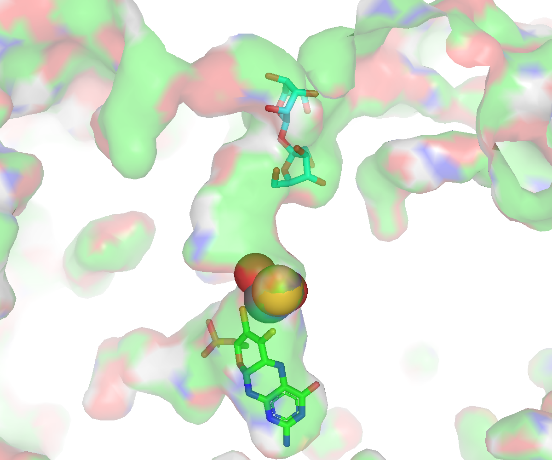 |
| BS-2 | OC[C@@H](O1)[C@@H](O)[C@H](O)[C@@H](O)[C@H]1O[C@@H](O2)[C@H](O)[C@@H](O)[C@H](O)[C@H]2CO | -1.96 | 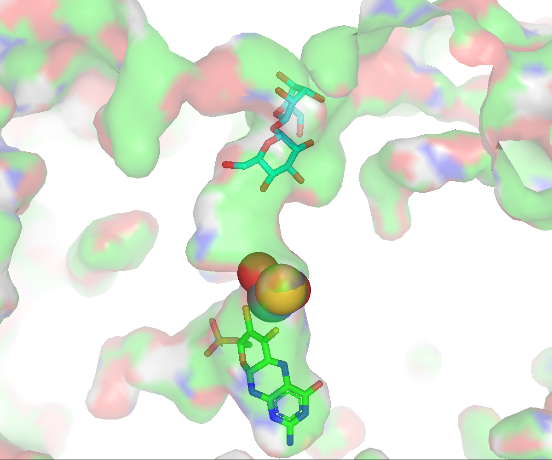 |
| BS-3 | O=C(O)C(O)CC(O)=O | -2.76 | 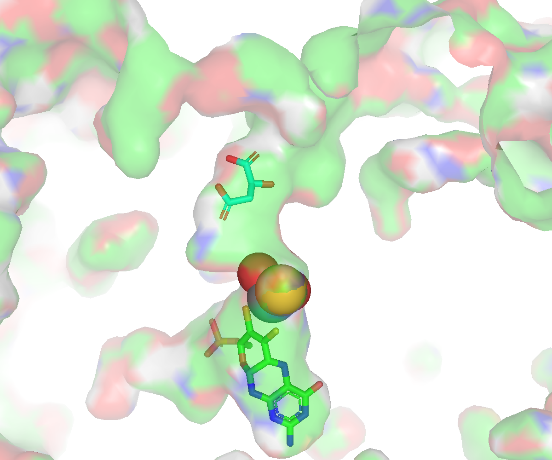 |
| BS-4 | OC1=C(O)C(O)=CC(C(O)=O)=C1 | -4.42 | 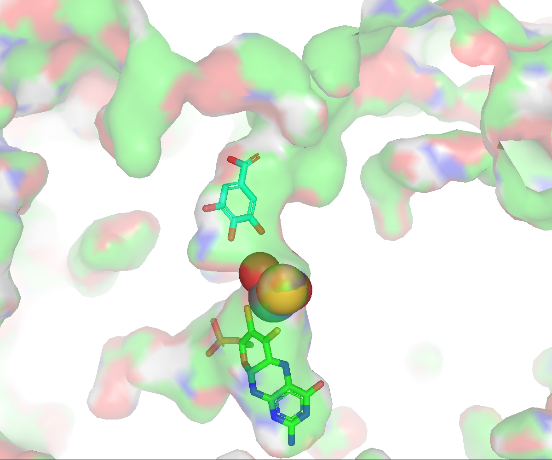 |
| BS-5 | OC1=C(O)C(O)=CC(C(OCC2C(O)C(O)C(O)C(OC(C3=CC(O)=C(O)C(O)=C3)=O)O2)=O)=C1 | -1.73 | 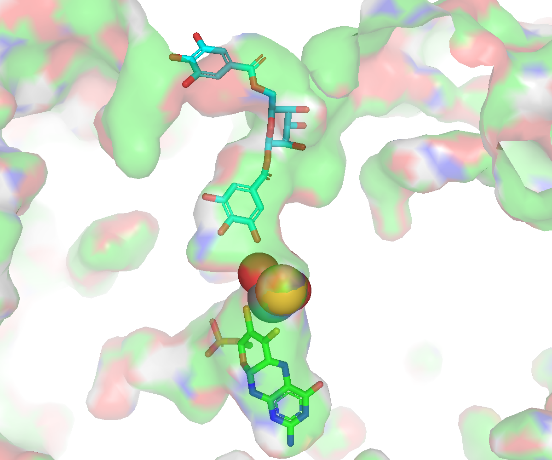 |
| BS-6 | O=C(C1=CC(O)=C(O)C(O)=C1)O[C@H](O2)[C@H](O)[C@@H](O)[C@H](OC(C3=CC(O)=C(O)C(O)=C3C4=C(O)C(O)=C(O)C=C45)=O)[C@H]2COC5=O | 7.29 | 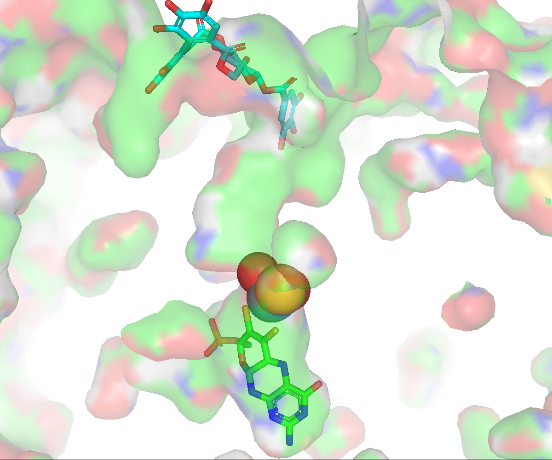 |
| BS-7 | O[C@H]([C@H]([C@@H]([C@@H](CO)O1)O)O)[C@@H]1OC(C2=CC=C(O)C(OC)=C2)=O | -3.71 | 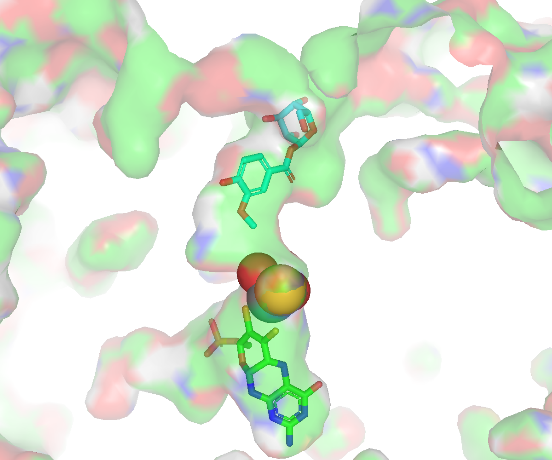 |
| BS-8 | O[C@H]([C@H]([C@@H]([C@@H](COC(C1=CC(O)=C(O)C(O)=C1)=O)O2)O)OC(C3=CC(O)=C(O)C(O)=C3)=O)[C@@H]2OC(C4=CC(O)=C(O)C(O)=C4)=O | 9.44 | 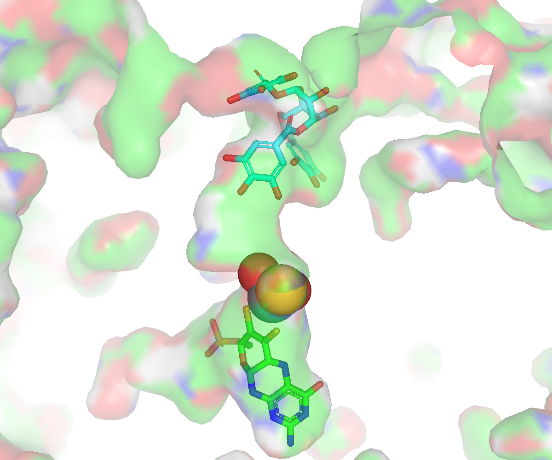 |
| BS-9 | O[C@@H]([C@@H]([C@@H](COC(C1=CC(O)=C(O)C(O)=C1)=O)O2)OC(C3=CC(O)=C(O)C(O)=C3)=O)[C@@H](OC(C4=CC(O)=C(O)C(O)=C4)=O)[C@@H]2OC(C5=CC(O)=C(O)C(O)=C5)=O | 65.30 | 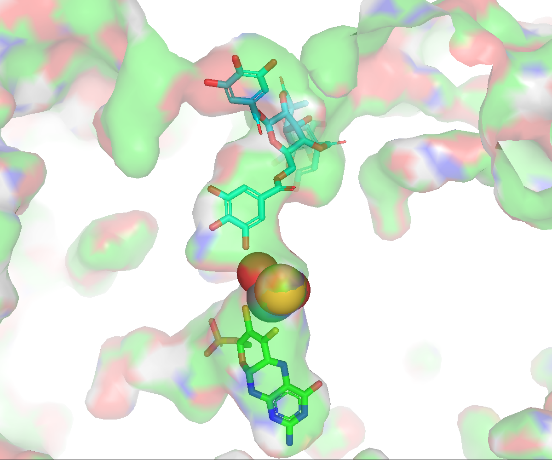 |
| BS-10 | OC(C(C(C(COC(/C=C/C1=CC=C(O)C=C1)=O)O2)O)O)C2OC(C3=CC(O)=C(O)C(O)=C3)=O | -3.62 | 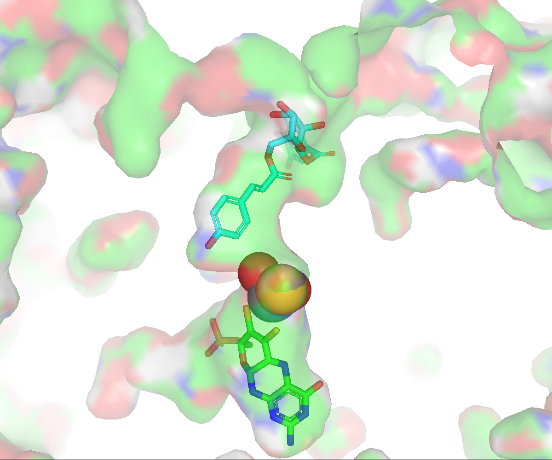 |
| BS-11 | O=C1CC(C2=CC=C(O)C(O)=C2)OC3=C1C(O)=CC(OC4[C@@H]([C@H]([C@@H]([C@@H](CO)O4)O)O)O)=C3 | -3.94 | 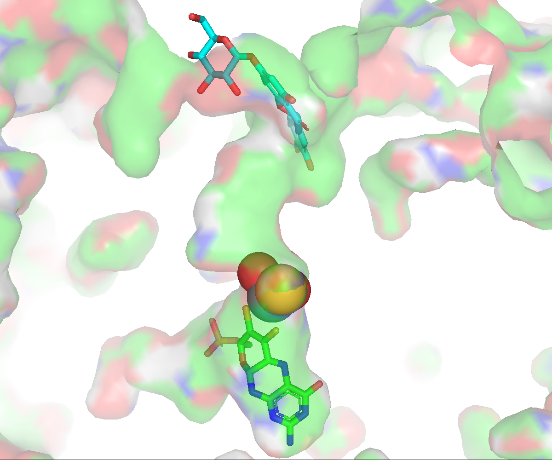 |
| BS-12 | COC1=C(O[C@H]2[C@H](O)[C@@H](O)[C@H](O)[C@@H](CO)O2)C=CC(C[C@H]3CO[C@H](C4=CC(OC)=C(O)C=C4)[C@H]3CO)=C1 | -3.41 | 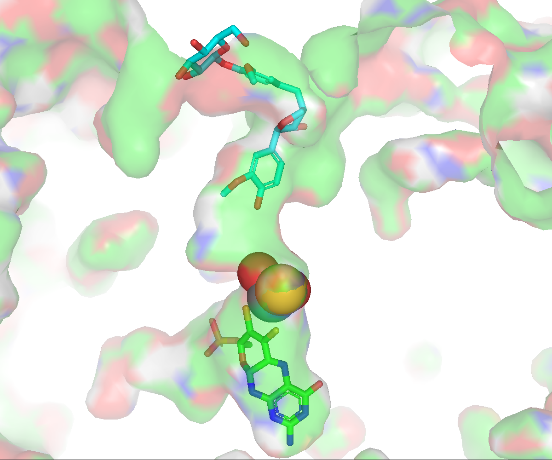 |
| BS-13 | O=C[C@@H]([C@H]([C@@H]([C@@H](COC(C1=CC(O)=C(O)C(O)=C1)=O)OC(C2=CC(O)=C(O)C(O)=C2)=O)OC(C3=CC(O)=C(O)C(O)=C3)=O)OC(C4=CC(O)=C(O)C(O)=C4)=O)OC(C5=CC(O)=C(O)C(O)=C5)=O | - | Too many torsion |
| BS-14 | O[C@@H]([C@@H]([C@@H](COC(C1=CC(O)=C(O)C(O)=C1)=O)O2)O)[C@@H](OC(C=CC3=CC=C(O)C=C3)=O)[C@@H]2OC(C4=CC(O)=C(O)C(O)=C4)=O | 0.44 | 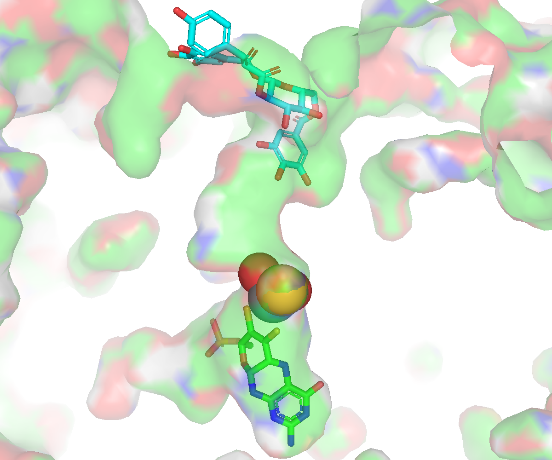 |
| BS-15 | O=C1C=C(C2=CC=C(O)C(O)=C2)OC3=C1C(O)=CC(OC4[C@@H]([C@H]([C@@H]([C@@H](CO)O4)O)O)O)=C3 | -3.97 | 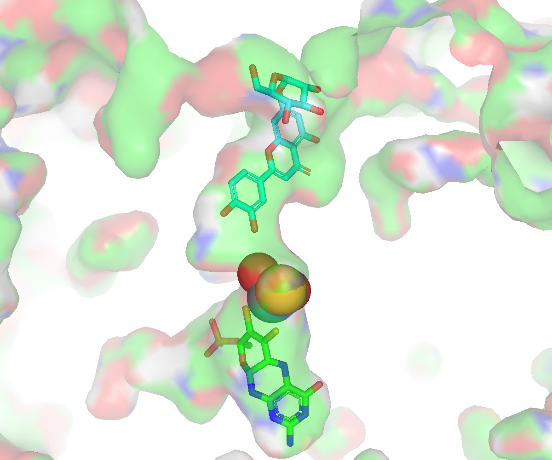 |
| BS-16 | COC1=C(O)C=C2[C@@H](C3=CC(OC)=C(O)C=C3)[C@@H](CO)[C@@H](CO)CC2=C1 | -4.71 | 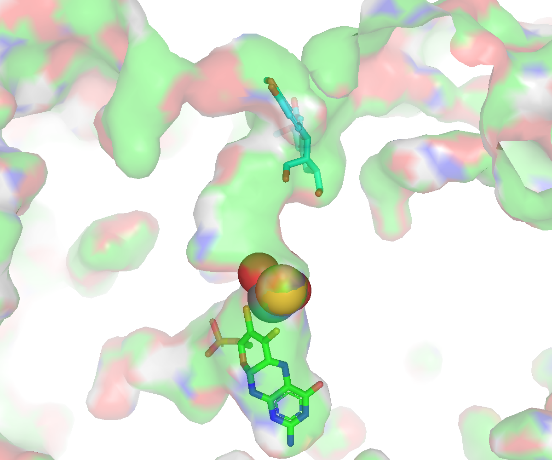 |
| BS-17 | O=C1OC2=C(C3=C1C=C(O)C(O)=C3OC4=O)C4=CC(O)=C2O | -5.17 | 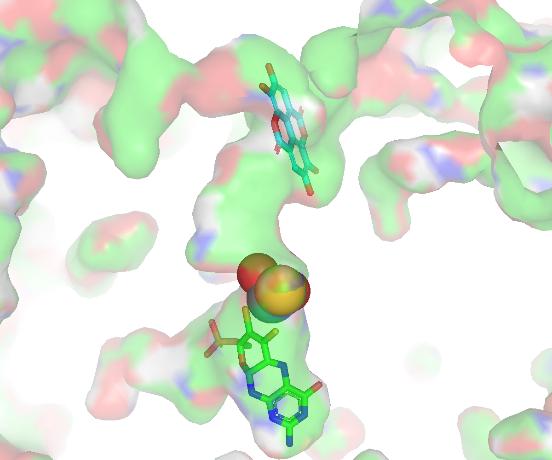 |
| BS-18 | O=C(O)[C@H](OC(/C=C/C1=CC(O)=C(O)C=C1)=O)CC2=CC(O)=C(O)C=C2 | -5.19 | 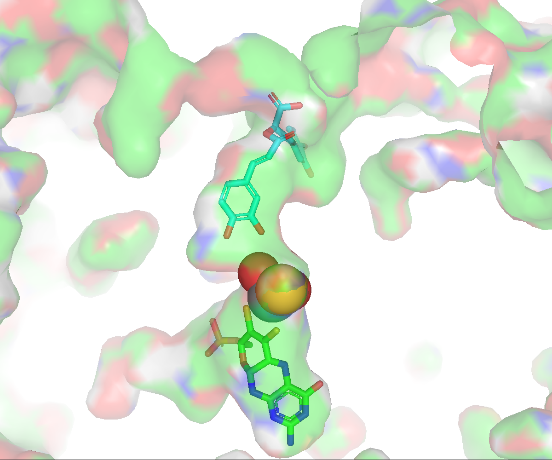 |
| BS-19 | O=C(O)CCCCCCCC(O)=O | -3.14 | 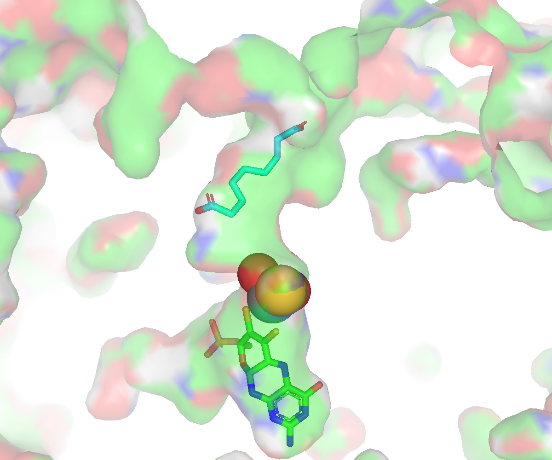 |
| BS-20 | OC1=CC=C(CCC(C2=C(O)C=C(O)C=C2O)=O)C=C1 | -6.62 | 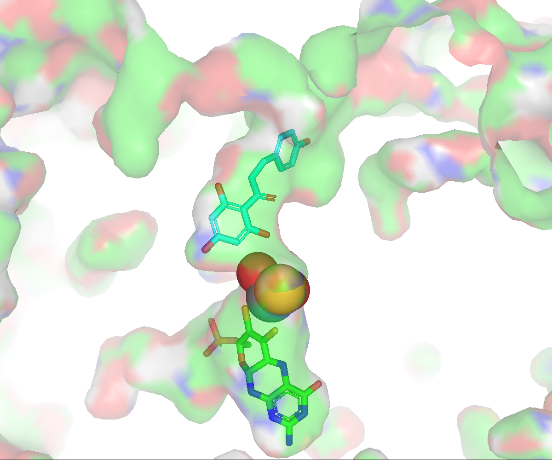 |
| BS-21 | O=C1CC(C2=CC=C(O)C=C2)OC3=C1C(O)=CC(O)=C3 | -6.84 | 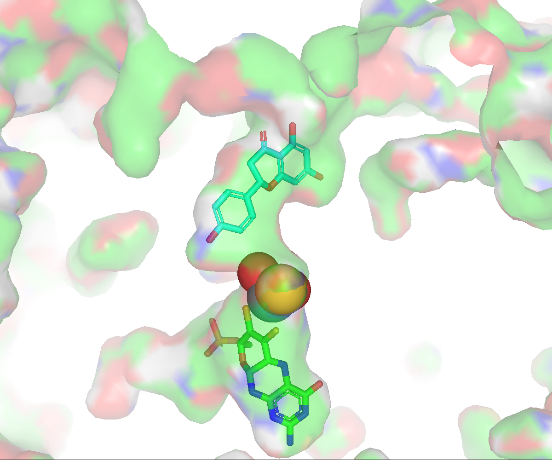 |
| BS-22 | O=C(C1=C(O)C=C(O)C=C1OC2C(O)C(O)C(O)C(CO)O2)CCC3=CC=C(O)C=C3 | -4.45 | 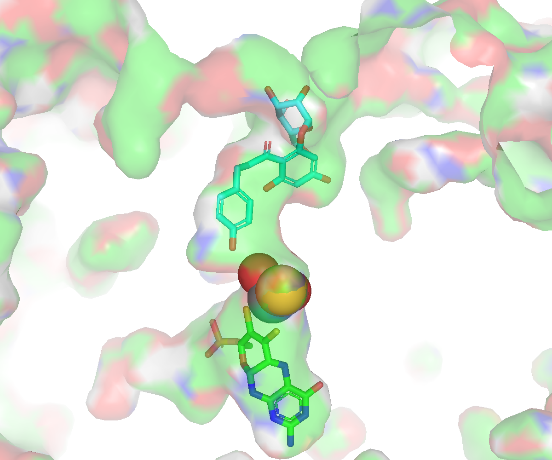 |
| BS-23 | O[C@H]([C@H]([C@]([C@@H](CO)O1)(C(C2=CC(O)=C(O)C(O)=C2)=O)O)O)[C@@H]1OC(/C=C/C3=CC=CC=C3)=O | -4.00 | 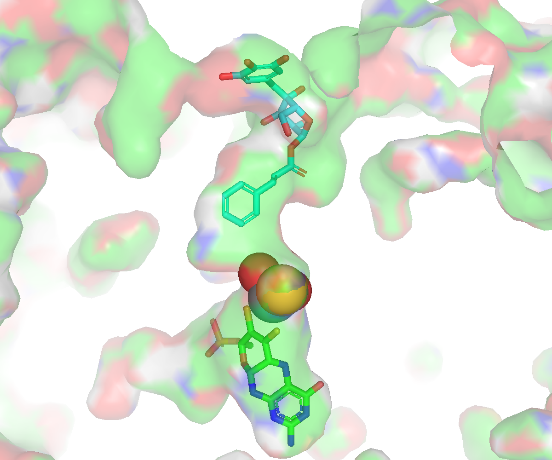 |
| BS-24 | CC12CC[C@H]3C([C@@H]1CCC24CCC(O4)=O)C=CC5=CC(CCC35C)=O | -5.20 | 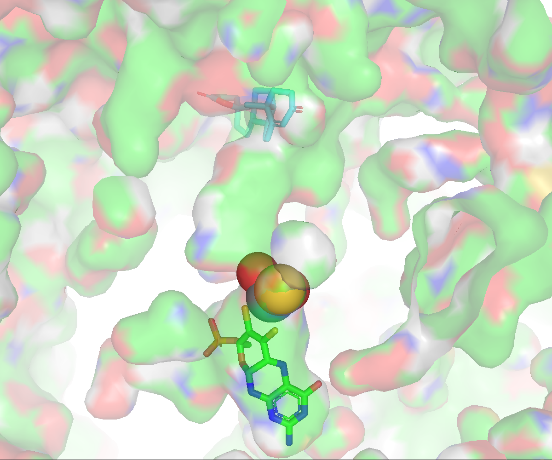 |
| BT-1 | O=C(C1=CC(O)=C(O)C(O)=C1)O[C@H](O2)[C@H](O)[C@@H](O)[C@H](OC(C3=CC(O)=C(O)C(O)=C3C4=C(O)C(O)=C(O)C=C45)=O)[C@H]2COC5=O | 23.26 | 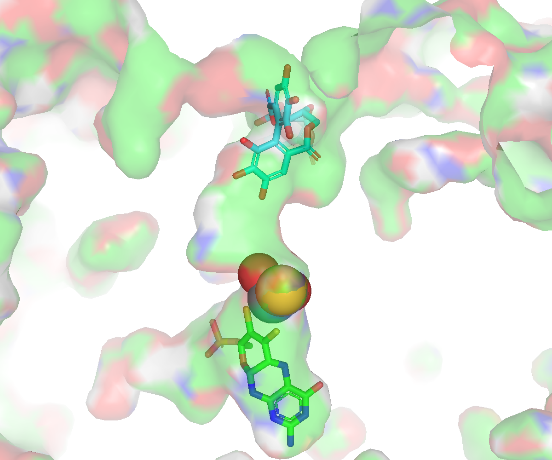 |
| BT-2 | OC1=C(O)C(O)=CC(C(OCC2C(O)C(O)C(O)C(OC(C3=CC(O)=C(O)C(O)=C3)=O)O2)=O)=C1 | -2.34 | 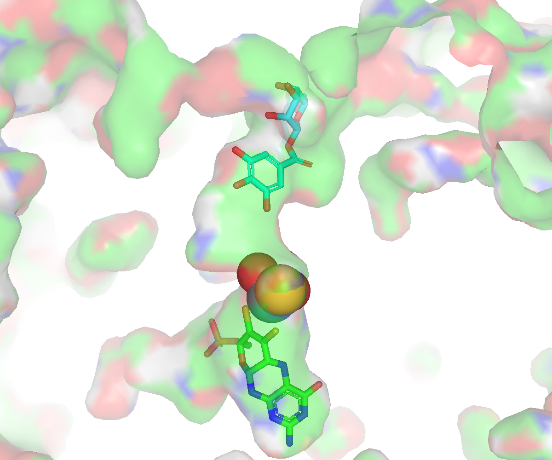 |
| BT-3 | O=C(C1C(C2=CC=C(O)C(O)=C2)C3=C(C=C(O)C(O)=C3)C=C1C(O)=O)O | -5.56 | 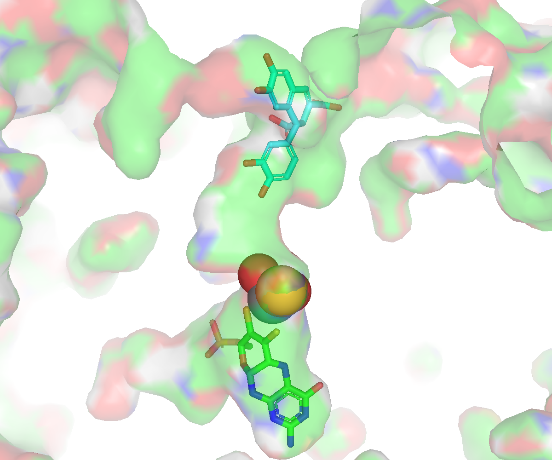 |
| BT-4 | C/C=C1/[C@H](O[C@@H]2O[C@H](CO)[C@@H](O)[C@H](O)[C@H]2O)OC=C(C(=O)OC)[C@H]1CC(=O)O[C@@H]1O[C@H](CO)[C@@H](O)[C@H](O)[C@H]1O | 4.25 | 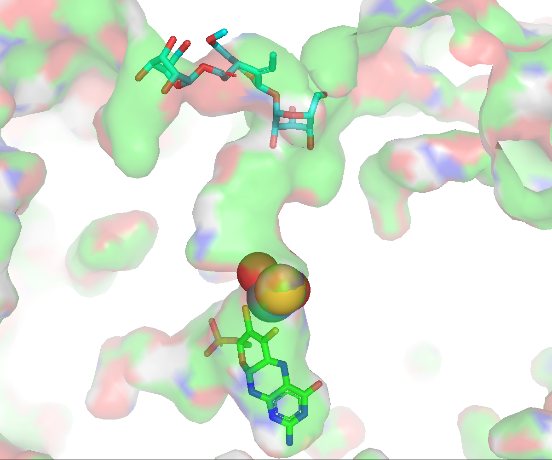 |
| BT-5 | O[C@H]([C@H]([C@@H]([C@@H](COC(C1=CC(O)=C(O)C(O)=C1)=O)O2)O)OC(C3=CC(O)=C(O)C(O)=C3)=O)[C@@H]2OC(C4=CC(O)=C(O)C(O)=C4)=O | -0.09 | 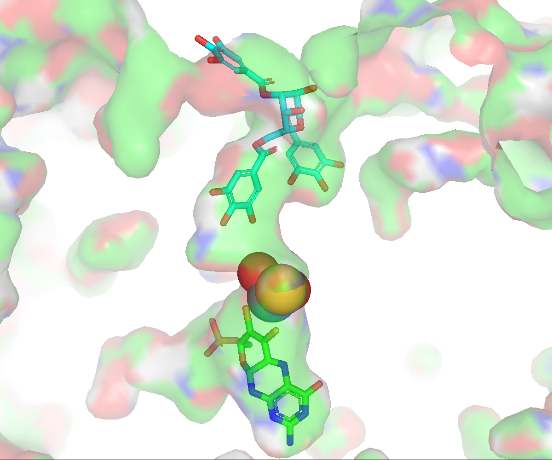 |
| BT-6 | COC(C1=CO[C@H]([C@@H]([C@@H]1CC(O)=O)C=C)O[C@@H]2O[C@@H]([C@H]([C@@H]([C@H]2O)O)O)CO)=O | -1.75 | 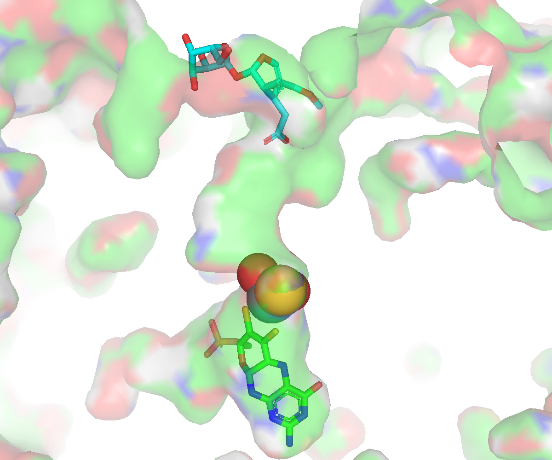 |
| BT-7 | O=C(C1O)C(C(O)=CC(O)=C2)=C2OC1C3=CC(O)=C(O)C=C3 | -6.46 | 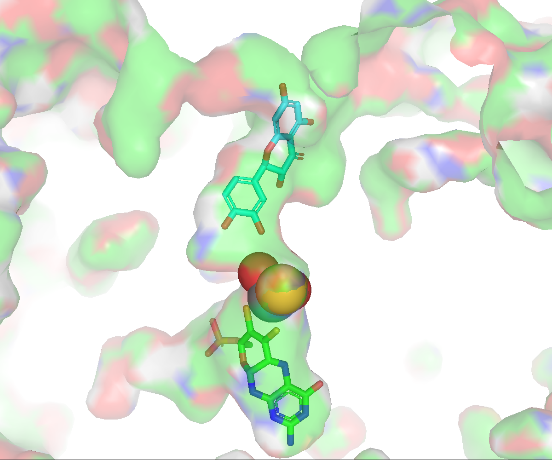 |
| BT-8 | COC1=C(O[C@H]2[C@H](O)[C@@H](O)[C@H](O)[C@@H](CO)O2)C=CC(C[C@H]3CO[C@H](C4=CC(OC)=C(O)C=C4)[C@H]3CO)=C1 | -2.30 | 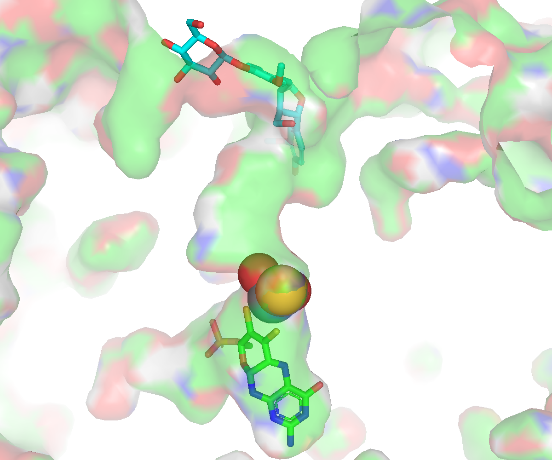 |
| BT-9 | O[C@@H]([C@@H]([C@@H](COC(C1=CC(O)=C(O)C(O)=C1)=O)O2)OC(C3=CC(O)=C(O)C(O)=C3)=O)[C@@H](OC(C4=CC(O)=C(O)C(O)=C4)=O)[C@@H]2OC(C5=CC(O)=C(O)C(O)=C5)=O | 47.28 | 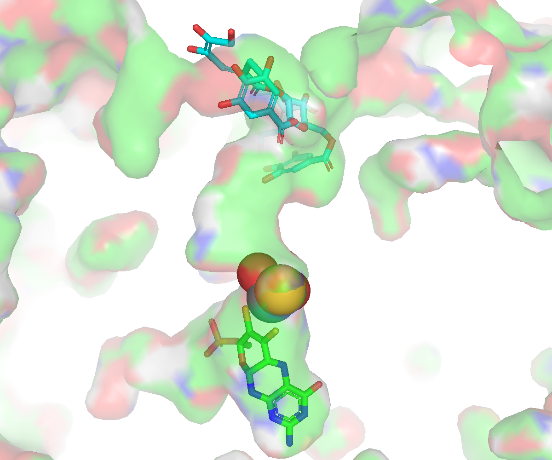 |
| BT-10 | O=C1CC(C2=CC=C(O)C(O)=C2)OC3=C1C(O)=CC(OC4[C@@H]([C@H]([C@@H]([C@@H](CO)O4)O)O)O)=C3 | -4.17 | 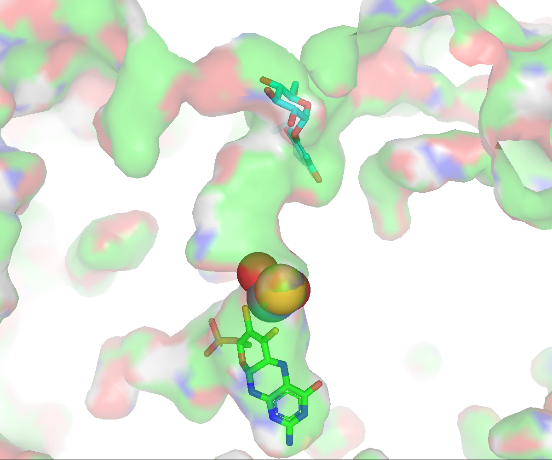 |
| BT-11 | O=C1C=C(C2=CC=C(O)C(O)=C2)OC3=C1C(O)=CC(OC4[C@@H]([C@H]([C@@H]([C@@H](CO)O4)O)O)O)=C3 | -4.26 | 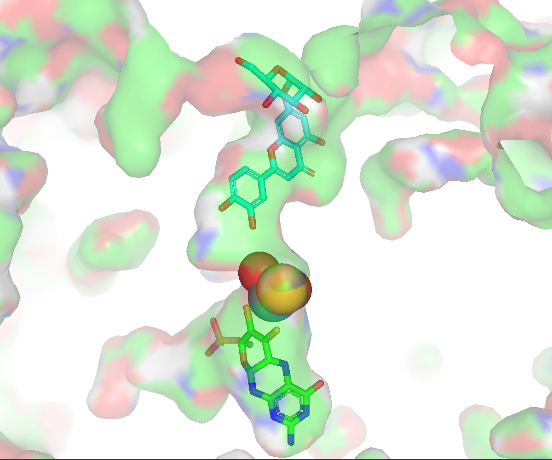 |
| BT-12 | OC1=C(C2=CC=C(O)C(O)=C2)OC3=C(C(O)=CC(O[C@H]4[C@@H]([C@H]([C@@H]([C@@H](CO)O4)O)O)O)=C3)C1=O | -5.20 | 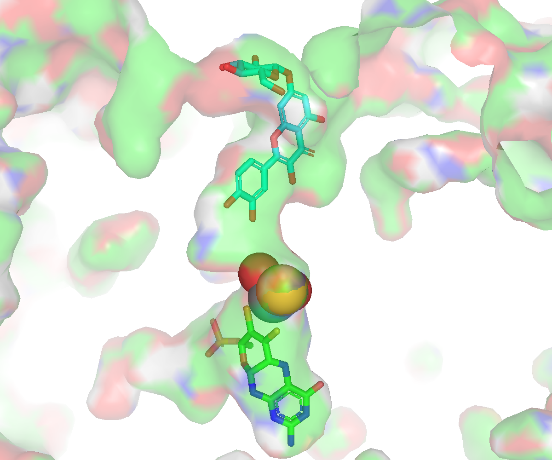 |
| BT-13 | COC1=C(O)C=CC(C[C@H]([C@H](CO)CC2=CC(OC)=C(O)C=C2)CO)=C1 | -3.61 | 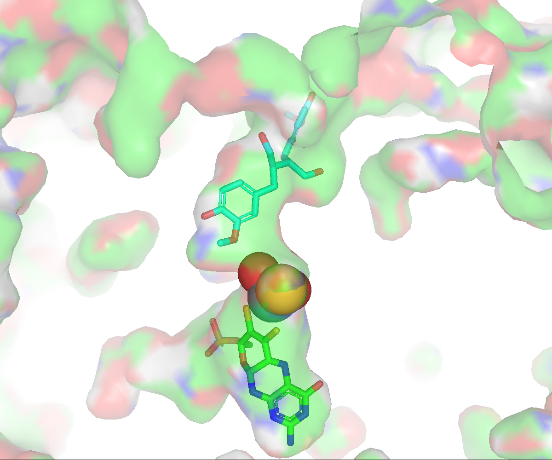 |
| BT-14 | O=C1CC(C2=CC=C(O)C=C2)OC3=C1C(O)=CC(O[C@H]4[C@H](O)[C@@H](O)[C@H](O)[C@@H](CO)O4)=C3 | -5.44 | 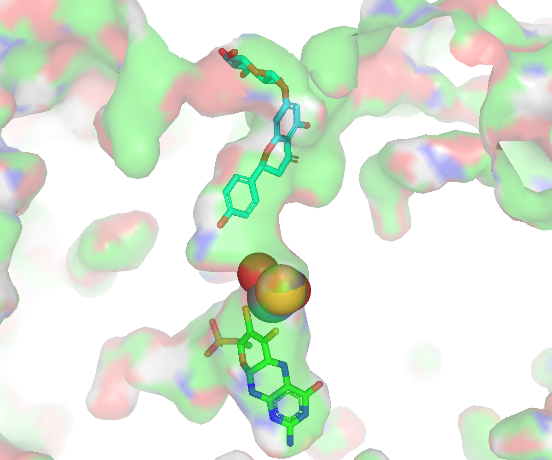 |
| BT-15 | O=C(O[C@H]1[C@H](Oc2c(-c3ccc(O)c(O)c3)oc3cc(O)cc(O)c3c2=O)O[C@H](CO)[C@H](O)[C@@H]1O)c1cc(O)c(O)c(O)c1 | 1.65 | 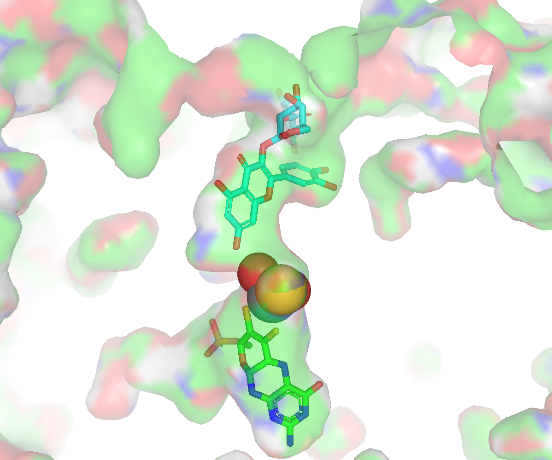 |
| BT-16 | O[C@H]([C@H]([C@]([C@@H](CO)O1)(C(C2=CC(O)=C(O)C(O)=C2)=O)O)O)[C@@H]1OC(/C=C/C3=CC=CC=C3)=O | -3.56 | 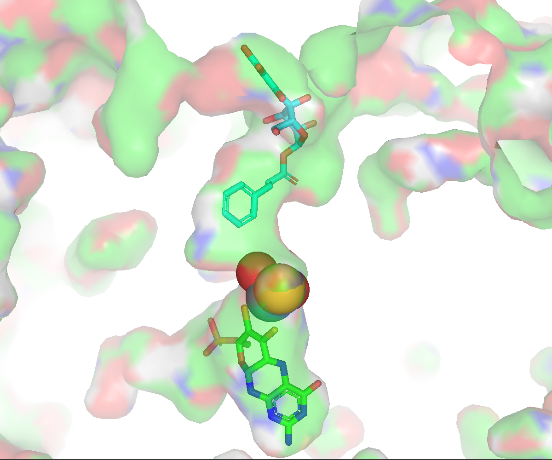 |
| BT-17 | O=C1OC2=C(C3=C1C=C(O)C(O)=C3OC4=O)C4=CC(O)=C2O | -5.15 | 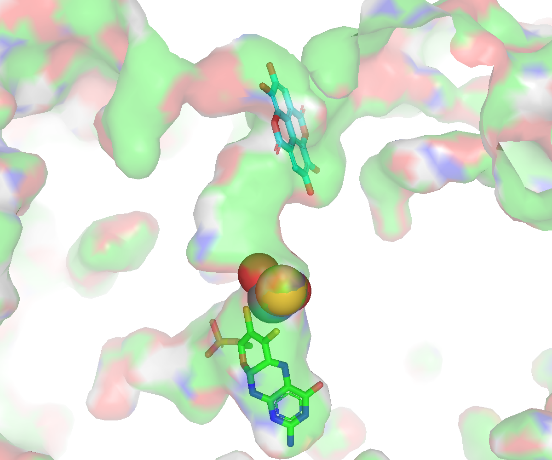 |
| BT-18 | OC1=C(O)C=C(CCOC(C[C@@]2([H])/C([C@H](OC3[C@@H]([C@H]([C@@H]([C@@H](CO)O3)O)O)O)OC=C2C(OC)=O)=C\C)=O)C=C1 | -0.47 | 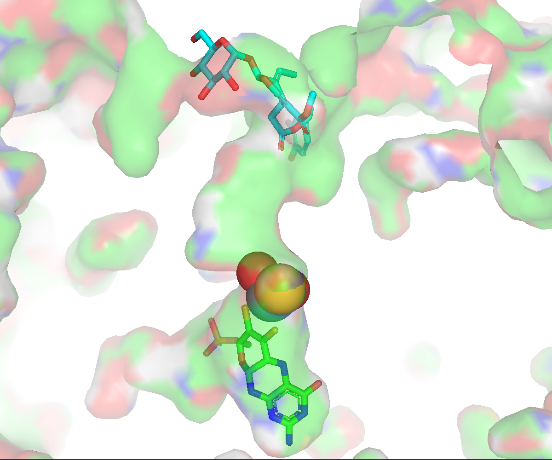 |
| BT-19 | O=C(C1=C(O)C=C(O)C=C1OC2C(O)C(O)C(O)C(CO)O2)CCC3=CC=C(O)C=C3 | -2.93 | 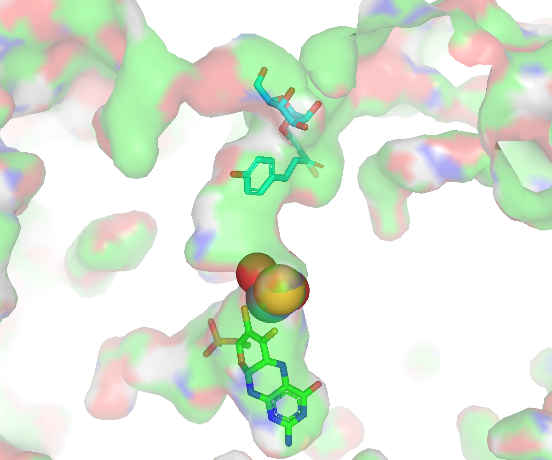 |
| BT-20 | O=C1CC(C2=CC=C(O)C=C2)OC3=C1C(O)=CC(O)=C3 | -6.85 | 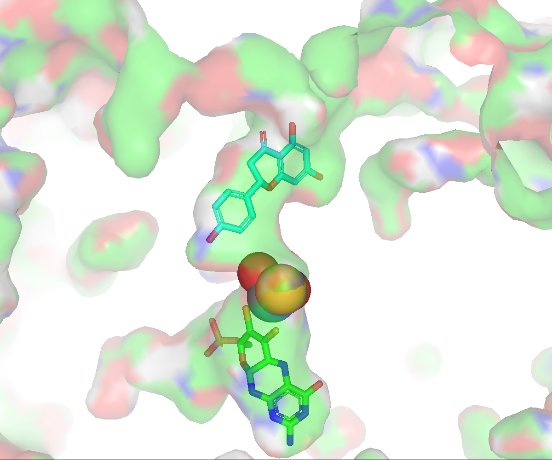 |
| BT-21 | O[C@H]1[C@@H](CO[C@@H]2O[C@H](CO)[C@@H](O)[C@H](O)[C@H]2O)O[C@@H](OC[C@H](COC(CCCCCCC/C=C\C/C=C\C/C=C\CC)=O)O)[C@H](O)[C@H]1O | 20.51 | 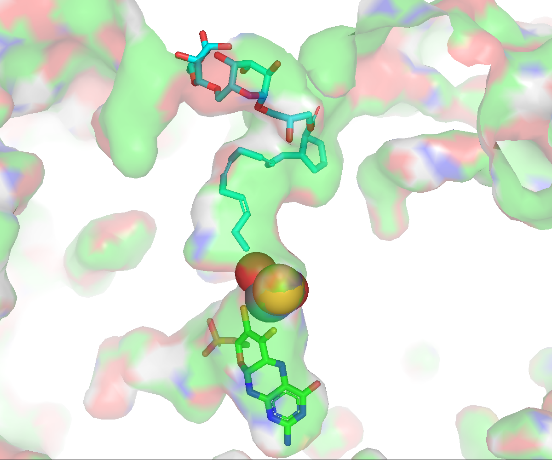 |
